# International Guidelines for the Treatment of Huntington's Disease

**DOI:** 10.3389/fneur.2019.00710

**Published:** 2019-07-03

**Authors:** Anne-Catherine Bachoud-Lévi, Joaquim Ferreira, Renaud Massart, Katia Youssov, Anne Rosser, Monica Busse, David Craufurd, Ralf Reilmann, Giuseppe De Michele, Daniela Rae, Ferdinando Squitieri, Klaus Seppi, Charles Perrine, Clarisse Scherer-Gagou, Olivier Audrey, Christophe Verny, Jean-Marc Burgunder

**Affiliations:** ^1^National Centre of Reference for Huntington's Disease, Henri Mondor Hospital, AP-HP, Creteil & NeurATRIS, Créteil, France; ^2^Clinical Pharmacology Unit, Instituto de Medicina Molecular, Lisbon, Portugal; ^3^IPMCN, School of Medicine, Cardiff University, Cardiff, United Kingdom; ^4^Centre for Trials Research, Cardiff University, Cardiff, United Kingdom; ^5^Division of Evolution and Genomic Sciences, Faculty of Biology, Medicine and Health, Manchester Centre for Genomic Medicine, School of Biological Sciences, University of Manchester, Manchester, United Kingdom; ^6^Manchester Academic Health Science Centre, Saint Mary's Hospital, Manchester University NHS Foundation Trust, Manchester, United Kingdom; ^7^Department of Radiology, George-Huntington-Institute, Universitaetsklinikum Muenster, Münster, Germany; ^8^Department of Neurodegenerative Diseases and Hertie-Institute for Clinical Brain Research, University of Tuebingen, Tuebingen, Germany; ^9^Department of Neurosciences, Federico II University, Naples, Italy; ^10^Department of Clinical Genetics, NHS Grampian, Aberdeen, United Kingdom; ^11^Huntington and Rare Diseases Unit, IRCCS Casa Sollievo della Sofferenza, San Giovanni Rotondo, Italy; ^12^Department of Neurology, Medical University Innsbruck, Innsbruck, Austria; ^13^Genetic Department, National Center of reference for Huntington's Disease, Salpêtrière Hospital, Paris, France; ^14^Neurology Department, Angers University Hospital, Angers, France; ^15^Neurology Department and UMR CNRS 6214 INSERM U1083, National Centre of Reference for Neurodegenerative Diseases, Angers University Hospital, Angers, France; ^16^NeuroZentrumSiloah and Department of Neurology, Swiss HD Center, University of Bern, Bern, Switzerland

**Keywords:** Huntington's disease, guidelines, treatment, care, clinical practice

## Abstract

The European Huntington's Disease Network (EHDN) commissioned an international task force to provide global evidence-based recommendations for everyday clinical practice for treatment of Huntington's disease (HD). The objectives of such guidelines are to standardize pharmacological, surgical and non-pharmacological treatment regimen and improve care and quality of life of patients. A formalized consensus method, adapted from the French Health Authority recommendations was used. First, national committees (French and English Experts) reviewed all studies published between 1965 and 2015 included dealing with HD symptoms classified in motor, cognitive, psychiatric, and somatic categories. Quality grades were attributed to these studies based on levels of scientific evidence. Provisional recommendations were formulated based on the strength and the accumulation of scientific evidence available. When evidence was not available, recommendations were framed based on professional agreement. A European Steering committee supervised the writing of the final recommendations through a consensus process involving two rounds of online questionnaire completion with international multidisciplinary HD health professionals. Patients' associations were invited to review the guidelines including the HD symptoms. Two hundred and nineteen statements were retained in the final guidelines. We suggest to use this adapted method associating evidence base–medicine and expert consensus to other rare diseases.

## Introduction

HD is a rare neurodegenerative disorder of the central nervous system, with a genetic autosomal-dominant inheritance, that first involves basal ganglia (caudate nucleus and putamen) and results from expansion of a CAG trinucleotide repeat in the HTT (huntingtin) gene: alleles with 40 or more repeats are fully penetrant. The disease is characterized by motor, cognitive and psychiatric disorders, and a range of somatic symptoms. Progressive worsening leads to a bedridden state with cognitive deterioration. Death occurs about 20 years after the onset of symptoms.

More than a century after the first description of Huntington's disease (HD), there is still no curative treatment of the disease; however, symptomatic treatments are thought to be efficacious in controlling some of its troublesome symptoms. Yet, symptomatic management of HD remains inadequately documented (1–4), which may lead to variations in care mainly based on clinical experience and not on scientific evidence (5–7).

This document provides scientifically supported and consensual pharmacological, surgical and non-pharmacological recommendations for the treatment of HD.

## Materials and Methods

### Methodology

**Figure 1:**
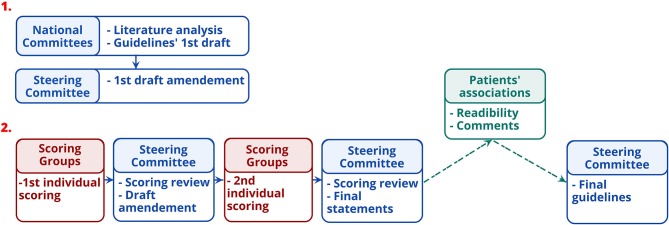
Guidelines' developing stages.

The EHDN guidelines task force developed guidelines between 2015 and 2018 based on a formalized consensus method, adapted from the 2015 French Health Authority recommendations (HAS) (https://www.has-sante.fr/portail/jcms/c_272505/recommandations-par-consensus-formalize-rcf). This method combines exhaustive review of the literature, experts' proposals, and external scoring of the proposals until agreement (Figure 1). This is particularly suitable when at least two of the following conditions are met (1) absence or insufficiency of high-level evidence specifically addressing the questions asked; (2) possibility of declining the theme in easily identifiable clinical situations; (3) controversy, with the need to identify by an independent group situation in which a practice is deemed appropriate. Its main advantages are (1) its ability to identify the degree of agreement or indecision among experts (2) the strict independence between the steering group, which formulates the proposals to be put to the vote, and the rating group which judges the appropriateness.

### Search Strategy

First, we conducted a search of scientific evidence published between 01/01/1965 and 01/08/2015 in the following databases: Cochrane Library, Embase, MEDLINE, PASCAL, BMJ Clinical Evidence, Current Contents, Infobanque AMC, National Guidelines Clearinghouse, PEDro, and BDSP (Public Health Database) as well as in the following websites: CEBAM, EBM sources, OMS Réseau de bases factuelles en santé, CBEM Oxford, Center for Evidence based child health, Center for health evidence, Center for reviews and Dissemination, Evidence based neurology, National institute for health and clinical excellence, Orphanet, ClinicalTrials.gov, OpenSIGLE (System for Information on Gray Literature in Europe). We also hand searched abstracts of international congresses of the Movement Disorders Society. Search terms were chosen based on a list of symptoms to focus on determined following discussions within the guidelines committee and working groups (neuroprotective, rehabilitation, and cognitive) of the European Huntington's Disease Network. Search terms were: “Huntington disease,” “drug therapy,” and symptoms (Huntington chorea, drug therapy, Chorea, Dystonia, Falls, Chokes, Bradykinesia, Rigidity, Depression, Apathy, Irritability, aggression, Obsessions, perseverations, Anxiety, Agitation, Hallucinations, delusions, paranoia, Impatience, Impulsivity, Suicidal Ideation, Memory, Loss of fluency, speech, Dysarthria, Attention disorders, Social cognition impairments, Disorientation, Bradyphrenia, Indecision, Weight loss, Incontinence, Sleep disorders, Diarrhea, Sweating, Constipation, Vomiting, Swallowing, Pain, Dental decay, and Surgery).

Drug manufacturers and authors were also contacted in order to obtain additional information on unpublished trials. In total 637 publications were collected.

### Data Extraction and Analysis

The Task Force committees reviewed the 637 collected publications with the French and UK committees focusing on pharmacological/surgical and non-pharmacological interventions, respectively. First, two members of each national committee conducted independently a screening of the collected publications and retained results from clinical trials, observational studies, meta-analysis, systematic reviews, case studies, previous recommendations, or conference and congress summaries. Studies including patients with HD clinical features and a confirmatory genetic diagnosis or a compatible family history (mostly for studies published before gene discovery in 1993) were also included 288 and 88 papers on pharmacological/surgical and non-pharmacological interventions, respectively, were retained for further analysis. The remaining members of the Task force validated the list of excluded publications. Second, a pair of members from each national committee summarized the key elements of the retained studies by filling a table with the following columns: authors, date of publication, type of intervention, daily dose (both of the active drug and the placebo), genetic characteristics of the patients (genetically diagnosed), study design, number of participants, duration of the study, primary and secondary endpoints, outcome, scales used, conclusion of the reviewers, and level of proof. Then they analyzed independently each study by assessing the methods (quality of the study) and results (the contents of the study) and assigned a level of scientific evidence according to the HAS classification (see below).

### Quality Appraisal and Data Synthesis

Following the HAS recommendations, a quality grade was attributed to each study according to the level of scientific proof they provided (Table 1) (Appendix 1).

**Table 1:** Level of scientific evidence and gradation of studies.

**Level of scientific proof provided by the study**	**Quality grade**
**Level 1** •Meta-analyses of randomized controlled trials •Randomized controlled trials of high power	**A** Established scientific proof
**Level 2** •Randomized controlled trials of low power •Properly conducted non-randomized controlled trials •Cohort studies	**B** Scientific presumption
**Level 3**Case-control studies	**C** Low-level of scientific proof
**Level 4** •Comparative studies with major bias •Case series	

### Method for Reaching a Consensus

The subsequent steps for developing the guidelines are displayed in Figure 1. First, the experts of the national committees formulated provisional recommendations for each HD symptom, classified in four categories of disorders (motor, psychiatric, cognitive, and “others”). Recommendations were based on the synthesis of information from the studies, i.e., quality grade, accumulation of scientific evidence, and professional expertise. Recommendations were rated according to the quality grades of the studies on which they are based, with the highest quality grade determining the score. When scientific evidence was lacking, best clinical practice (professional agreement) was formulated, based on the experience of the National committees. The International Steering Committee reviewed the initial recommendations before initiating process to reach a consensus with the International Multidisciplinary HD Health Professionals group (Table 2). This involved two rounds of online questionnaire completion. After the first round, only appropriate recommendations with strong consensus were retained (Table 3). Those without strong consensus were reviewed and modified by the International Steering Committee prior to the second round of ranking (Table 3). After the second round, all recommendations were deemed appropriate, and agreed as such, except two of the motor chapter and two of the psychiatric chapter. Two hundred and nineteen statements were retained in the final guidelines. The steering committee added a rider considered important by the multidisciplinary group to the four recommendations that did not reach a consensus. Whereas, the literature basis scored through survey monkey ends in 2015, experts' and knowledge input were provided through the survey scoring and comments as well as the last face-to-face meeting until October 2018.

**Table 2:** Composition of the international multidisciplinary HD health professionals.

		**Motor disorders**		**Cognitive disorders**		**Psychiatric disorders**		**Other disorders**	
		**1st survey**	**2nd survey**	**1st survey**	**2nd survey**	**1st survey**	**2nd survey**	**1st survey**	**2nd survey**
Number of participants		67	38	63	36	60	32	56	30
Expertises	Dentists	1	1	0	0	0	0	0	0
	Geneticists	4	3	3	2	2	0	3	1
	Neurologists	39	24	35	20	34	20	33	22
	(Neuro)psychologists	8	2	11	6	10	4	7	2
	Nurses	3	3	2	2	2	2	3	2
	Physiotherapists	5	1	4	2	4	1	3	1
	Psychiatrists	7	4	8	4	8	5	7	2
	Countries	Australia Belgium Brazil Chile Cyprus Czech R. Denmark Estonia France Germany Hungary Italy Netherlands Peru Poland Portugal Romania Russia South Korea Spain Sweden Switzerland UK USA	Australia Brazil Chile Cyprus Czech R. Denmark Estonia France Germany Hungary Italy Netherlands Poland Portugal Russia Spain Switzerland UK USA	Australia Belgium Brazil Chile Cyprus Czech R. Denmark Estonia France Germany Italy Netherlands Poland Portugal Romania Russia Spain Switzerland UK USA	Australia Belgium Brazil Chile Cyprus Czech R. Denmark Estonia France Germany Italy Netherlands Poland Portugal Romania Russia Spain Switzerland UK USA	Australia Belgium Brazil Chile Cyprus Czech R. Denmark Estonia France Germany Italy Netherlands Peru Poland Portugal Romania Russia Spain Switzerland UK USA	Australia Brazil Chile Cyprus Czech R. Denmark Estonia France Italy Netherlands Peru Poland Portugal Romania Russia Spain Switzerland UK USA	Australia Belgium Brazil Chile Cyprus Czech R. Denmark Estonia France Germany Italy N. Zealand Netherlands Peru Poland Portugal Romania Russia Spain Switzerland UK USA	Australia Brazil Chile Czech R. Denmark Estonia France Italy Netherlands Peru Poland Portugal Romania Russia Switzerland UK USA

**Table 3:** Rules to determine the strength of the consensus of the multidisciplinary experts.

**Recommendations deemed as**		**1st round**			**2nd round**	
		**Median value**	**Responses' distribution**	**Submitted to the 2nd round**	**Median value**	**Responses' distribution**
Appropriate	Strong agreement	≥7	All between (7-9)	No	≥7	All between (7-9); two values missing or <7 tolerated
	Relative agreement	≥7	All between (5-9)	Yes	≥7	All between (5-9); two values missing and/or <5 tolerated
Inappropriate	Strong disagreement	≤3	All between (1-3)	No	≤3	All between (1-3); two values missing or >3 tolerated
	Relative disagreement	≤3.5	All between (1-5)	Yes	≤3.5	All between (1-5); two values missing or >5 tolerated
Indeterminate	Indecision	Between [4–6.5]	Whatever the distribution	Yes	Between [4–6.5]	Whatever the distribution
	No consensus	≥7	At least one <5 or missing	Yes	≥7	At least three <5 or missing
		≤3.5	At least one >5 or missing	Yes	≤3.5	At least three >5 or missing

### Patients' Associations Involvement

European, Chinese and French HD associations as well as the Italian League for Research on Huntington and related diseases Foundation were invited to review the guidelines.

## Results

A condensed version of HD symptoms and recommendations is provided in the main text. A full version is available in Appendix 2. Publications justifying the grades of the recommendations are cited in the text. Recommendations provided without specific grading are underpinned by professional agreements.

Given that any HD symptoms may be worsened by stress, fatigue, and intercurrent disorders (e.g., anxiety, digestive disorders, infectious or painful conditions, etc.), these aspects must be assessed and should be treated with appropriate measures alongside managing the Huntington's symptoms.

### Motor Disorders

The wide spectrum of motor manifestations are the best known and the most visible symptoms in HD. Among them, involuntary movements (i.e., chorea) are the most obvious. However, while the diagnosis of manifest HD is based on the presence of motor symptoms, these are frequently preceded by cognitive and behavioral symptoms (8). While motor symptoms are easily detected, and might be the source of anxiety and ostracism, they are often well-tolerated by the patients and their proxies in contrast to cognitive and behavioral symptoms that often lead to family and social/professional's issues.

#### Chorea

Chorea is characterized by abnormal, involuntary, spontaneous, uncontrollable, irregular, intermittent, non-rhythmic and aimless movements affecting the trunk, the face, and the limbs.

Drug treatment should be considered if chorea causes the patient distress or discomfort.

Tetrabenazine is one of the first-line treatments for this symptom (Grade A) (9) unless the patient suffers from not well-managed depression or suicidal thoughts. Second generation neuroleptics (Grade B) (10, 11) are first-line treatments for this symptom in particular when the patients have associated personality and/or behavioral or psychotic disorders. Monotherapy to treat chorea is preferred because combination therapy increases the risk of adverse effects and may complicate the management of non-motor symptoms. In the presence of disturbing chorea, appropriate protective measures (especially during meal times and during the performance of instrumental activities of daily living) should be put in place to avoid traumatic injury or chokes. Rehabilitation specialists can help identify appropriate assistive technology devices and positioning techniques.

#### Dystonia

Dystonia is characterized by abnormal postures that may affect all body segments and is frequently associated with rigidity (12). Dystonia intensity varies from a slight intermittent abnormal posture to severe twitch of muscles with major impact on movements and functions of daily living.

Both active and passive physiotherapy approaches are recommended as a preventive measure to maintain the range of joint motion, limit postural and musculoskeletal deformities and, prevent the development of contractures. Injection of botulinum toxin in the case of focal dystonia or to prevent secondary deformities should be performed by a trained professional. Customized chairs can provide a comfortable environment in view of the dystonia-related deformities.

#### Rigidity

Rigidity is an increase in muscle tone leading to a resistance to passive movement that can induce joint stiffness and limited range of motion, which might be distressing for patients.

Rigidity may be increased or induced by the use of neuroleptics or tetrabenazine. If this impacts the functional capacity of the patient, a reduction in dosage or the withdrawal of neuroleptics and/or tetrabenazine should be considered considering overall benefit on chorea and/or behavioral symptoms vs. severity of rigidity.

Levodopa may provide partial and temporary relief of the akinetic–rigid symptoms of HD, especially in juvenile forms (Grade C) (13–18). Treatment with levodopa should be started gradually and the total daily dose is usually lower than in Parkinson's disease.

Physiotherapy is recommended to improve or maintain mobility and prevent the development of contractures and joint deformity (Grade C) (19).

#### Akathisia

Akathisia is a syndrome characterized by unpleasant sensations of “inner” restlessness that manifests as an inability to sit still.

An iatrogenic cause of akathisia should be investigated as the priority.

Tetrabenazine (Grade C) (20, 21), neuroleptics and Selective serotonin reuptake inhibitors (SSRI) may cause akathisia in HD and reducing the dose or changing the treatment may be helpful.

#### Swallowing Disorders

Swallowing disorders can occur in patients at the early stages of the disease and become a major problem in later stages by inducing repeated choking and leading to secondary bronchopulmonary infections or even cardiac arrest.

Regular assessment of swallowing disorders should be provided throughout the progression of the disease (Grade C) (22) and referral to a Speech and Language Therapist is recommended as soon as the disorders appear (Grade C) (22–24).

Ancillary assessments that may help in managing swallowing disorders include: generalized motor skills, respiratory status, dental health, mood, behavior and emotional status, cognition, nutrition, and hydration status. Provision of information and advice on safe swallowing procedures, on posture and positional changes can help to avoid aspirations and leads to improvement of swallowing disorders. Oral-facial exercise with swallow sequence individualization and cough post swallow may also improve swallowing difficulties. In some cases, treating chorea might help in improving swallowing problems. However, side effects of treatments for chorea (e.g., sedation, attention, and parkinsonism) might also negatively impact swallowing capacities.

The education of carers is important as they are often managing the eating, drinking, and swallowing regime.

For severe swallowing disorders impacting nutrition and quality of life of the patient, the use of a gastrostomy device Percutanous Endoscopic Gastrostomy (PEG) may be considered and should be discussed on a case-by-case basis with the patient and the caregivers. PEG should be anticipated and discussed with relatives and patients still able to understand the benefits and burdens of the methods. Before advanced stages of the disease, patients should be educated to make an informed choice concerning the PEG methods even if they can change their decision at any time.

#### Myoclonus

Myoclonus refers to sudden muscle contractions, brief and involuntary, axial, in extremities or generalized, similar to spams and jerks in epileptic seizures but not related epilepsy. In HD, myoclonus can be observed in a predominant akineto-rigid phenotype and can be associated with an at rest or action tremor, especially in the juvenile forms but also in later-onset forms. In juvenile forms, non-epileptic myoclonus can coexist with epilepsy.

In case of myoclonus impacting the functional capacity of the patients, treatment with sodium valproate or clonazepam, used alone or in combination, and in escalating doses, is recommended (Grade C) (25–32). Levetiracetam is a therapeutic alternative for the same indication. In case of myoclonus of cortical origin that is not associated with epileptic seizures, piracetam has a marketing authorization (Grade C) (29). Benzodiazepines, in particular clonazepam, may be used to manage myoclonus whilst remaining vigilant with regard to adverse effects such as somnolence and increasing falls, and the risk of drug-dependence.

#### Gait and Balance Disorders

Gait and balance disorders impairments include disruption of cadence regulation, increased variability of step width and length, disturbed initiation and increased postural sway (33). These develop as a result of the progressive complex movement disorder seen in HD adding to the overall burden of motor morbidity with falls and loss of independence in HD (34).

Generally, interventions for gait and balance should start as early as possible and be continued and adapted throughout the progression of the disease (Grade C) (33, 35–38). Physiotherapy interventions (Grade B) (39–42) and the introduction of falls prevention programs, gait, core stability, and balance interventions (Grade C) (35, 43–45) as well as attentional training are recommended.

Pharmaceutical management of chorea may improve walking and balance as they can be affected by chorea (Grade C) (46–49). However, they should always be used cautiously and regularly reassessed as their adverse effects may also aggravate walking disorders.

Maintaining physical activity and low impact exercises is recommended.

The use of assistive devices such as four-wheeled walker (Grade B) (50) as recommended by Physiotherapist or Occupational Therapist should be considered to improve stability and reduce fall risk.

#### Bruxism

Bruxism is an involuntary clenching with excessive contraction of the jaw muscles. It typically causes lateral movements (or front to back) responsible for gnashing and can lead to tooth damage.

Injecting botulin toxin A into the masseter muscles is proposed as the first-line treatment of bruxism (Grade C) (51). Customized protective mouth guards may be used to reduce the complications of bruxism on a case-by-case basis, mostly in early stage patients.

Bruxism may occur as a side effect of neuroleptics (Grade C) (51, 52) and serotonin reuptake inhibitors, thus reducing their dose should be considered.

#### Manual Dexterity

Manual dexterity can be impaired secondary to chorea/dystonia/akinesia/rigidity but also occur in their absence—due to abnormal motor planning and sequencing.

Neuroleptics and tetrabenazine may possibly have a beneficial effect on dexterity as a result of reducing chorea (Grade C) (46, 47, 53) but may also have a detrimental effect on dexterity by aggravating other symptoms such as bradykinesia.

Management with physiotherapy and occupational therapy may be useful to reduce the functional impact of fine motor skill deterioration (Grade B) (41). Adaptive aids may help to compensate for the deterioration of manual dexterity.

#### Global Motor Capacities

Early referral to a physiotherapist is recommended in order to facilitate the development of a therapeutic relationship, promote sustainable exercise behaviors and ensure long-term functional independence.

Physiotherapy and/or personalized exercise programs (Grade B) (40) are beneficial for the overall functional ability, motor function, and independence in HD, in combination with pharmacological treatments (Grade B) (39, 40, 42).

#### Cognitive Disorders

Cognitive deficits appear frequently before motor symptoms (8). They are, in addition to behavioral symptoms, the major cause of family disruption and social withdrawal (54). Cognitive symptoms cause intense psychological discomfort and a sense of powerlessness that can lead to behavioral symptoms.

Based on present knowledge, no pharmacological treatment is recommended for the treatment of cognitive symptoms.

Multiple rehabilitation strategies (speech therapy, occupational therapy, cognitive and psychomotricity) might improve or stabilize transitorily cognitive functions at some point of time in the course of the disease (Grade B) (55).

#### Executive Functions

Executive functions refer to the functions that allow the realization of complex task in daily living. They consist in a set of functions mostly dedicated to cognitive and behavior control and adaptation, which may be impaired in HD, even at the premanifest stages and thus impose adaptation from the environment, organization support including proactivity in planning appointments, behavior or daily life activities like cooking.

For the patients to maintain their independence for as long as possible, it is better to help the patients organize themselves and initiate activities rather than substitute for them, as long as they do not endanger themselves.

Treatment for anxiety and depression may help to improve executive function and cognitive stimulation through rehabilitation may improve planning and initiation more specifically (Grade C) (56). Sedative drugs and neuroleptics should be closely monitored as they impair executive functions and attention.

#### Bradyphrenia

Bradyphrenia is defined by slowing of cognitive information processing and a prolongation of reaction time depending on the complexity of the cognitive task (57). It becomes more apparent with HD disease progression.

Management is based on giving the patient enough time to process information and perform a task and avoiding time-pressured situations. Cognitive stimulation as part of rehabilitation may be beneficial.

#### Language and Communication Disorders

Language and communication disorders can be divided in speech and language disorders *per se*. Speech disorders consist of slurred and slowed speech causing dysarthria, inappropriate pauses or bursts of speech, and progressive reduction in verbal fluency (58). Language (e.g., syntax) impairments appears early in the disease course, with progressive difficulties in understanding and producing complex sentences. Reduction of lexical capacities appears later. This often goes unnoticed and may cause misunderstanding and impaired communication.

The changing communication needs of the person with HD should be reassessed throughout the course of the disease to plan effective management strategies (Grade C) (59). As communication disorder in HD is variable, its monitoring requires comprehensive assessment of language and of other factors such as mood, motivation, and behavior.

Early referral to Speech and Language Therapists is recommended (Grade C) (59) as they can play a major role in assessing and managing communication problems in HD at all stages of the disease. Communication strategies and techniques may include: management options (e.g., voice therapy techniques), advice on facilitation of communication (e.g., allowing time for communication, reduction of environmental distractions and noise) and the use of simple technics (e.g., gestures and rephrasing) or tools (e.g., pen and phones).

Family members and other communication partners should be educated to support patients to attempt verbal communication as long as possible. Augmentative and alternative communication (talking mats) can compensate for communication difficulties and increase the individual's chance of participation in daily life. These strategies need to be implemented whilst there is still motivation and a capacity to learn (Grade C) (60).

#### Social Cognition Impairments

Social cognition impairments refer to a set of symptoms that affect relationships and social behavior. The most studied are the inability to recognize emotion others (61) but also to express emotions, both through facial expression or through the voice. Executive function impairments can make difficult for the patients to express their feelings. The capacity to infer other thoughts or feeling, are also reported to be impaired in patients (61). Furthermore, motor impairments can create a “facial mask,” often misunderstood as indifference.

Improvement of behavioral disorders may help with social and family integration. However, impact of SSRI or neuroleptics on social interaction *per se* has not yet been properly assessed to allow any recommendation specific to this domain.

Explaining the patients' disorders to their family, health care professionals or to their colleagues may facilitate the patient's social relationships. Moreover, third party intervention (e.g., caregiver, nurse, and social worker) may stimulate patients' social interaction.

#### Memory Disorders

Memory disorders are frequently reported in HD and may be confounded with or exacerbated by attention disorders. They are mostly characterized by difficulties in learning new information and retrieving information acquired (62).

Strategies such as establishing and keeping a regular daily routine may compensate memory loss. Rehabilitative approaches (speech therapy or neuropsychology) may help memory as part of an overall intervention plan. Specifically, domain-specific transcoding (verbal and visual) may help in recalling items.

Sedative drugs, neuroleptics and tetrabenazine may impact negatively on memory.

#### Disorientation

Disorientation, both in time and space, appear during the progression of HD but temporal orientation is altered earlier (63–66).

Investigations should be carried out to detect any potential intercurrent cause for a confusional state. Establishing a regular routine, in tune with the patient's environment as much as possible, and milestones enables the patient to manage their time better.

#### Visuospatial and Visual Perceptual Disorders

Visuospatial and visual perceptual disorders appear late in the course of the disease through interference with the integration and understanding of visual information (66).

It may be useful to make the patient's environment safe (padding furniture) to minimize falls and shocks linked to visual spatial difficulties and aggravated by motor disorders.

#### Psychiatric Disorders

Behavioral symptoms may appear before the motor diagnosis of the disease. They are, in addition to and in conjunction with cognitive symptoms, the major cause of family disruption, social isolation, and withdrawal.

Their management should be based on the identification of the underlying triggers causing changes in mood or behavior. Patients should be given the opportunity to express their worries and frustrations.

Using methods to calm and reassure patients is a major component of care of psychiatric disorders. Based on data from other neurodegenerative conditions, mindfulness-based cognitive therapy and Acceptance and Commitment Therapy may be useful in HD.

#### Depression

Depression is one of the most common psychiatric symptoms seen in HD (67, 68) with a significant negative impact on quality of life. It may affect patients at any stage of the disease, even before motor manifestation (69). Thus, vigilance to detect and treat depression is required at all stages of the disease.

Psychotherapy and cognitive behavioral therapy may enable early detection of mood changes. An antidepressant may be suggested if depression occurs in HD (Grade B) (70). It is recommended to use a selective SSRI or a serotonin noradrenaline reuptake inhibitor (SNRI), or alternatively Mianserin or Mirtazapine, in case of sleep disruption. In case of recurrent depression, long-term mood-stabilizer treatment may be introduced in complement to the treatment of the current episode to prevent relapses. If depression is thought to be an adverse effect of other medication, the dosage of the responsible drug should be reduced gradually. In the case of resistant depression, or depression associated with psychotic symptoms, a psychiatrist should be consulted. In case of severe depression and resistant to oral medications, electroconvulsive therapy (ECT) may be suggested under the guidance of a psychiatrist (Grade C) (71–73).

#### Suicidal Ideation or Attempts

Suicidal ideation or attempts are common in HD (74) and correlate with family history of suicide, a history of previous suicide attempts and the presence of depression, especially in prodromal stages (75).

Suicide risk should be assessed in HD irrespective of the stage, being particularly vigilant at the time of diagnosis and when the disease starts to impact on day-to-day life. Prevention of suicide includes treating risk factors such as underlying depression, social isolation and impulsivity.

#### Irritability

Irritability is a very common symptom in HD (67, 68, 76). This disorder is of fluctuating nature, characterized by impatience and a tendency to become angry in response to minimal provocation. Overflow and loss of control are favored by impulsivity, and can lead to aggressive behavior toward self or others, and rarely, to criminal behavior. This symptom can be caused by the frustrations felt by the patient because of the great loss of his capacities, and by troubles in expressing himself, as well as by neurological/psychological fatigue brought by the latter.

Before initiating pharmacological treatment, possible environmental causes for the patient's frustration and irritability should be explored. In order to reduce irritability, behavioral strategies should be considered. A structured plan with a regular routine in a calming environment is desirable. In addition, psycho-education for the patient's family regarding diversion strategies should be attempted to avoid confrontation as much as possible.

Whilst SSRIs are first lines for irritability (Grade C) (77, 78), it may be necessary to use them at or near the maximum recommended dose in order to be effective. Irritable patients who do not benefit from an SSRI alone may benefit from combination therapy with Mianserine or Mirtazapine, especially when sleep disorders are present. In patients with aggressive behavior, the recommended first-line treatment is a neuroleptic (Grade C) (79–81). In case of overt aggression associated with depression, neuroleptic treatment should be associated with sedative antidepressants. If irritability does not respond to antidepressant therapies and/or neuroleptics, a mood stabilizer (Grade C) (82, 83) can be added.

#### Apathy

Apathy has been defined by Levy and Czernecki (84) as “a quantifiable reduction in goal-directed behavior,” manifesting clinically as a reduction in interest, spontaneity, motivation, and drive. In patients with HD it is compounded by emotional blunting, resulting in social withdrawal, and lack of concern for others. It is the most frequent psychological and behavioral symptom in HD, especially in the middle and later stages, causing a severe reduction in the activities of daily living and often being a source of conflict in the family. With regard to cognitive and psychological symptoms, apathy and irritability are the two faces of the same coin (85). A patient can be apathic the morning and irritable the afternoon, depending on the situation. As for irritability, apathy can be caused by environmental and psychological issues. Apathy may also be an adaptive response when the patient feels overwhelmed by too much stimulation (HD patients are more sensitive to noise and environmental interferences), or with the feeling that his/her disease is progressing.

It is important to explain the various aspects and causes of the apathy to the family circle.

Personalized cognitive stimulation, establishing routines and a structured programme of activities is recommended when possible. A professional intervention at home can improve compliance and reduce the patient's opposition and irritability.

Depression may increase apathy. If depression is suspected, an SSRI should be tried.

Sedative medication may increase apathy, thus avoiding unnecessary prescription or reduce dosage is recommended.

#### Anxiety

Anxiety as defined by the uncomfortable feeling of nervousness or worry about something that is happening or might happen in the future, is common in HD. Anxiety is linked to the other symptoms (motor and cognitive), as the patient is anxious because of the loss of essential functions, and correlated to family, social and economic issues, and to the burden of his pathology (and the one of his proxies). However, anxiety does not increase with disease progression. It is associated with depression, suicide, irritability, quality of life, pain, illness beliefs, and coping.

SSRI or SNRI are first line treatments of anxiety, especially when associated with depression. On-demand prescription of an anxiolytic might be beneficial, but caution is required because of the associated risk of worsening or causing falls. Neuroleptics (Grade C) (86, 87) are valuable therapeutic alternatives in the treatment of anxiety when other treatments fail.

#### Obsessions

Obsessions are defined by recurrent and persistent thoughts, ideas or images that do not let the mind rest, causing anxiety. True obsessions, according to this definition, are not very common in patients with HD, but perseveration is very common, particularly in the middle and later stages (76). Perseveration may be defined as the repetition of a thought, behavior or emotion beyond the psychological context in which it arose, and in patients with HD these repetitive thoughts and behaviors can persist for hours, months, or even years after the original trigger. Patients have little or no insight into the problem (in contrast to obsessional thoughts, which are distressing and recognized as abnormal); however, it has been shown that perseveration is the one behavioral symptom in HD which has a significant negative impact on the quality of life of family members and caregivers (88).

Over the course of HD, symptoms may change and repetitive thoughts may replace obsessive–compulsive disorder. The distinction between obsessive–compulsive phenomena and perseverations is important for the care strategy, both requiring differential approaches.

If pharmacological treatment is necessary for perseverative symptoms, an SSRI could be prescribed (Grade C) (89), in particular when symptoms are associated with anxiety. Olanzapine and risperidone (Grade C) (81, 86) are two valuable therapeutics for ideational perseverations, in particular when they are associated with irritability.

True obsessive–compulsive phenomena are sensitive to psychological intervention, such as Cognitive Behavioral Therapy, in non-cognitively impaired patients. If pharmacological treatment is necessary for obsessive-compulsive phenomena, a SSRI should be prescribed as first-line treatment (Grade C) (89).

#### Impulsivity

Impulsivity consists of acting without prior planning, which can lead to unpredictable behavior. When impulsivity is associated with depression or irritability, there is a significant increased risk of self-harm or suicide or aggressiveness. Impulsivity may be the result of cognitive impairments, which lead to an intense frustration toward patience, the patient being in the incapacity to wait or to deal cognitively with planning. Impulsivity may then be an adaptive response to language difficulties of patients who cannot explain what stresses them.

When impulsivity is associated with depression or personality disorders, there is a risk of auto- or hetero-aggressiveness, which justifies the prescription of a neuroleptic in combination with a SSRI. Long-term mood-stabilizer treatment may be introduced in the case of mood lability and impulsivity.

#### Sexual Disorders

Sexual disorders are very common in HD. Decreased libido is the most common symptom while hypersexuality or disinhibited behavior are rarer, but can cause significant problems in relationships. Repetitive hypersexual behaviors are often a result of perseveration.

Identifying the existence of sexual disorders and determining their triggers and their impact on relationships is important. Psychological support and/or referral to a specialist in psychosexual disorders might be useful. In the case of decreased libido, an iatrogenic cause should be investigated (e.g., the use of an SSRI) and reducing the dose or substituting the treatment responsible may be suggested. In the case of erectile dysfunction, treatment for impotence may be suggested and seeking the opinion of an endocrinologist and/or a specialist in psycho-sexual disorders may be useful. In case of impotence, prescription of phophoesterase 5 inhibitors should be considered in the clinic when asked for by the patient and his sexual partner. A behavioral and psychological approach is useful in the case of hypersexuality, by re-establishing appropriate standards of behavior in the patient's social setting. If hypersexuality involves social discomfort or violence, the proposed first-line treatment is a neuroleptic (Grade C) (90) and/or a SSRI. If the treatment for hypersexuality with neuroleptics and/or SSRI is not successful, the addition of or substitution for an anti-androgen may be proposed (Grade C) (91–93) under the guidance of a specialist in sexual disorders or an endocrinologist. Where hypersexuality poses a risk to others, specific measures should immediately be put in place (e.g., referral to a psychiatrist).

#### Hallucinations

Hallucinations are defined as a perception without an object, at which the subject adheres to and reacts as if the perception came from outside. Delusions are false beliefs based on incorrect inferences about external reality, the cultural and social context to which the patient belongs.

The use by the patient of psychotropic agents should be searched for and interrupted in case of hallucinations and delusions. Second generation neuroleptics are the first line treatment for hallucinations and delusions (Grade C) (80, 81, 86, 94–106). Clozapine should be proposed as the first-line treatment in the case of akinetic forms of HD with debilitating Parkinsonian symptoms. Perseverative ideation can sometimes mimic psychotic symptoms, and in such circumstances the patient may benefit from treatment with serotoninergic antidepressants in combination with an atypical neuroleptic. Psychiatric intervention and support are particularly useful in the case of psychotic disorders occurring in HD, for treatment adjustments. If pharmacological treatments fail, the option of ECT can be discussed with psychiatrists (Grade C) (71, 73, 107).

In case of agitation, priority should be given to identifying environmental or somatic triggers (bladder distension, fecal impaction, pain, etc.) in order to treat the underlying cause, especially in the advanced stages of the disease when communication difficulties exist. When agitation is associated with an anxiety disorder, a benzodiazepine should be prescribed as needed to reduce the risk of dependence and falls (Professional agreement). Some benzodiazepines (e.g., midazolam) may be useful in emergency situations. Long-term treatment with benzodiazepines should be avoided as much as possible but remains necessary in some patients. In the case of extreme agitation, and if there are associated behavioral and personality disorders, it is advised to prescribe a neuroleptic (Grade C) (82, 90, 91, 102, 108, 109).

#### Other Disorders

Other symptoms than motor, cognitive and psychiatric disorders are often present. Among those, weight loss, dysphagia, and sleep disturbance are not unfrequently the most prominent symptoms. As they may cause discomfort, they should be looked for in order to limit them when present.

#### Sleep Disorders

Sleep disorders are common in HD. Around two-thirds of HD patients suffer from sleep disorders, with diverse causes such as depression, anxiety, intrinsic alteration in the circadian sleep-wake rhythm, and involuntary movements during sleep inducing awakenings (110, 111). They may present as difficulties in falling asleep and/or early awakenings in the middle of the night followed by insomnia. They may be associated with aimless wandering, and lead to difficulties in coping by the proxies. However, disturbances of diurnal rhythm (day-night reversal, etc.) are probably more common than simple insomnia in HD patients.

Potential underlying cause of sleep-related difficulties (e.g., depressive syndrome, anxiety, and severe involuntary movements) should be investigated. Simple lifestyle and dietary strategies (e.g., avoiding long nap, having no stimulants after 4 pm) are the first-line treatment of insomnia. When lifestyle strategies are ineffective to treat insomnia, prescribing a hypnotic may be suggested for a short duration to avoid the risk of drug dependence. Some agents may be proposed in place of a hypnotic and for a long duration (e.g., mianserin, mirtazapine, and antihistaminic drugs) as they have a reduced tendency for causing dependency. Melatonin may be suggested in case of sleep phase inversion. A neuroleptic should be prescribed in the evening when sleep disorders are associated with behavioral disorders or chorea.

#### Urinary Incontinence

Urinary incontinence may either be multifunctional or linked to a deterioration of the frontal lobe control centers, causing an overactive bladder with urge incontinence and/or unannounced urination (112).

Where there is urinary incontinence, a precipitating factor should always be investigated (urinary infection, prostate disease). It is useful to investigate the presence of diurnal unexpected complete urination (complete and sudden bladder emptying, without urge) for which carbamazepine may be of benefit (Grade C) (112). In the case of an overactive bladder with leakage and urge incontinence, therapy with selective antimuscarinic may be tried, whilst watching out for the appearance of potential side effects, in particular confusional state. If, after few weeks, the incontinence therapy has not been effective, it should be stopped. If simple therapeutic measures have failed, it is advised to undergo urodynamic testing to help guide the choice of drug therapy and to consult a urologist if necessary.

In all cases, it is recommended to implement simple lifestyle strategies: urination before every outing and at regular times.

#### Pain

Pain assessment is sometimes difficult because of communication disorders. Moreover, because of communication's disorders and a tendency for these patients not to complain, pain is often related to non-verbal language and behavioral disorders such as irritability and restfulness.

Behavioral change or worsening of involuntary movements should trigger the search for an underlying source of discomfort, and in particular pain.

#### Dental Pain

Patients suffer from poor oral health for a variety of reasons, including impaired motor ability (e.g., difficulties brushing teeth) or reduced motivation to maintain oral health, the use of drugs affecting salivary secretion and frequent dental trauma due to falls and injuries, bruxism.

Multidisciplinary teamwork, especially with dietitians to avoid highly cariogenic foods, is recommended (Grade C) (113, 114). Verbal and written instructions on how to provide good oral hygiene at home should be given to patients and carers (Grade C) (114, 115). Dental care including descaling by a dentist or dental hygienist should be carried out at least once a year but should be more frequent in the later stages of the disease.

At later stages of the disease, treatment options should be discussed carefully and in advance. Treatment intervention, especially in late stage disease may require conscious sedation (midazolam, Diazepam) or general anesthesia in a hospital setting (Grade C) (115–117).

In view of the frequency of digestive disorders in HD (e.g., constipation, diarrhea, and vomiting) and their impact on the quality of life of patients, routine assessment for these symptoms is recommended in order to ensure their management.

Their diagnostic workup should be conducted by the relevant specialists (general and digestive examination, biological and radiological tests, scan, fibroscopy, colonoscopy, etc.). Fecal impaction should be routinely investigated where there is constipation/ diarrhea (“false” diarrhea) and/or vomiting. Vomiting is sometimes intractable. If no specific etiology is identified, the following should be considered: staggering meals, reviewing the patients' posture during and after the meal, and possibly reducing antichoreic agents, in particular neuroleptics.

#### Excessive Perspiration

Excessive perspiration can occur at all stages of HD. It can be associated with other autonomic disorders and reflects discomfort or emotional burst when sudden.

In the case of excessive perspiration, care must be taken to ensure patients are well-hydrated, monitored and that their fluid and electrolyte balance is adjusted. Thyroid function and the possibility of infection should be assessed in case of excessive perspiration.

#### Weight Loss

Weight loss is often present in HD, sometimes prior to the appearance of other symptoms. It might occur despite normal, or even high calorie intake, due to a significant energy expenditure in HD patients. It can also be caused by swallowing disorders, depressive syndrome with reduced appetite or gastrointestinal disturbance and gut abnormalities due to enteric neuron dysfunction (118).

Good nutritional care is a fundamental element of the management of HD (Grade C) (119, 120). Early assessment by a dietitian or nutritionist, and regular timely reviews of nutritional needs are recommended. Factors such as swallowing ability, cognitive changes, behavior, mood, and general functional ability should be considered to determine possible other causes of weight loss (Grade C) (23, 120–123). A multi-disciplinary approach is recommended and may include a Speech Language Therapist and an Occupational Therapist to assist with swallowing, positioning and feeding aids. Screening tools for malnutrition [e.g., malnutrition Universal Screening Tool (MUST)] are recommended.

A high Body Mass Index (BMI) within normal values should be maintained if possible and medical and/or social intervention is recommended when unintended weight loss is higher than 10% within last 3–6 months or when BMI is <20 kg/m^2^ and unintentional weight loss of 5% is observed within last 3–6 months. When weight loss is observed, high-calorie and high-protein food supplements should be prescribed under instruction and monitored by a dietician/nutritionist (Grade C) (124, 125).

A Mediterranean diet may improve Quality of Life and nutritional composition (Grade C) (126).

In case of the initiation of antidepressant and/or neuroleptic treatments, treatments inducing weight gain should be preferred in patients with significant weight loss, whilst treatments inducing weight loss should be avoided (these effects can vary from one patient to another) (Grade C) (127).

Advanced care planning is essential and alternative feeding methods (PEG, see swallowing disorders) should be anticipated and discussed with relatives and patients still able to understand the benefits and risks of the intervention.

#### Hypersalivation

Hypersalivation can be troublesome in HD patients when associated with a salivary incontinence (caused by poor oral occlusion and or fault swallowing).

In the absence of a specific treatment for HD, drugs used in other chronic diseases may be considered to reduce salivary secretion: scopolamine given percutaneously, atropine given orally or other drugs that have an anticholinergic effect (amitriptyline), whilst watching out for iatrogenic risks, in particular confusional state, constipation, ocular hypertension and urinary retention. Injections of botulinum toxin into the salivary glands may be considered in a specialized setting if oral or oral mucosa treatment options have not induced benefit or were not well-tolerated.

#### Reduced Lung Function and Respiratory Muscle Strength

Reduced lung function and respiratory muscle strength are not only associated with end stage disease but occur much earlier, with evidence of some upper airway changes in pre-symptomatic individuals and reduction of cough effectiveness, reduced lung volume, and impaired respiratory strength by mid-disease. Along with changes in posture reduced exercise capacity, these impairments negatively impact respiratory function, leaving patients vulnerable to respiratory infections.

Home-based respiratory muscle training program appeared to improve pulmonary function in manifest HD patients but had only a small effect on swallowing function, dyspnea, and exercise capacity (Grade B) (128).

## Conclusion

The EHDN guidelines task force provides here scientific and consensual guidelines from experts from 15 European experts from the national and steering committees and 73 worldwide additional experts from 25 countries. Whereas, the literature extraction and scoring extent from 1965 to 2015, experts' input extended until October 2018. To ensure the validity of the guidelines in the light of the latest scientific results, two authors reviewed the literature from 2015 to 2019. They extracted 573 abstracts and selected the 17 relevant studies to HD management, which were then added to the grids. Two authors analyzed them separately and assigned each of them a level of scientific evidence. Because these recent relevant studies were not used to formulate recommendations reviewed by the International Multidisciplinary HD Health Professionals group, they are mentioned in the conclusion. Except for deutetrabenazine (Grade A) (129, 130), none of the studies justified to modify the recommendations. Deutetrabenazine may indeed be proposed as an alternative to tetrabenazine for the treatment of chorea in countries where the marketing authorization is already obtained, like in the USA. In addition, a number Grade B and C studies were in agreement with the current recommendations and reinforce the interest of rehabilitation (131–135). Therefore, as they stand, with this precision, these guidelines are likely to serve as international for care in HD. They are likely to support both general practitioners and specialists' decisions. Patients associations and patients themselves may use them and also disseminate them to inform their doctors.

It becomes increasingly clear that the cost of health is one of the major issues of public policy. In countries where there is a medical insurance system, the question of the choice of therapeutic care or medication and rehabilitation in the insured basket constitutes a central issue. The difficulty is even greater in rare diseases such as HD because the number of patients is too small to carry out double-blind placebo-controlled studies on large cohorts (Grade A) as required for the selection of health policies according to evidence-based medicine. In this work, based on therapeutic trials conducted between 1965 and 2015, only one grade A study was found among 376 studies analyzed, which is insufficient to eliminate or recommend enough products to meet the patients' needs. In parallel, thanks to specific international networks dedicated to HD (EHDN, HSG, and ERN) experts' know-how has increased with a knowledge-learning culture over time. In this context, the French Ministry of Health has labeled Rare Diseases Reference Centers in 2004, imposing on them various duties, one of which is producing National Protocols for Diagnostics and Care (NPDC). These protocols are designed as a combination of comprehensive literature reviews and expert consensus combining the work of an expert panel, and then its validation by outside experts to compensate for the information that is lacking. The recommendations from these NPDCs made it possible to provide decision-makers with comprehensive information based on an adapted version of evidence-based medicine to rare diseases. In addition, they allowed the health professional to refer to a document to answer their questions of day-to-day care. EHDN, with more than 2,000 members in 50 countries, is concerned by the relevance of prescriptions, medical procedures, hospital stays, care pathways, and care arrangements. It thus commissioned an international adaptation of the French NPDC. To give it an international value, we replaced face-to-face meetings with electronic votes and added international committees and patient associations to national committees. Thus, beyond offering international guidelines to practitioners for the management of HD, this document proposes a method that is likely usable in all rare diseases.

## Author Contributions

A-CB-L supervised the elaboration of the guidelines. OA, KY, CS-G, and RM selected the studies to be analyzed. A-CB-L, KY, CP, CS-G, and DR analyzed each study and assigned a level of scientific evidence. Members of the National Committees (A-CB-L, CV, KY, CP, CS-G, OA, DR, and DC) formulated initial recommendations for each HD symptom. Members of the Steering Committee (A-CB-L, JF, KY, AR, MB, DC, RR, GD, DR, FS, KS, and J-MB) reviewed the initial recommendations and supervised the writing of the final recommendations. RM supervised the online surveys, analyzed the results, and assisted the Steering Committee in the writing of the recommendations. Members of the Steering Committee and RM wrote the manuscript (original draft preparation, review, and editing).

### Conflict of Interest Statement

J-MB was chair of the European Huntington's Disease Network (EHDN) during the completion of the work, and received honoraria for the duties involved in this position. AR received reimbursements as Co-Chair and Chair of the EHDN. RR is the owner of the George-Huntington-Institut GmbH and the QuantisMedis GmbH. He is a member of the EHDN Executive Committee and of the Huntington Study Group Executive Committee. The remaining authors declare that the research was conducted in the absence of any commercial or financial relationships that could be construed as a potential conflict of interest.

## European Huntington's Disease Network Guidelines Task Force

**• Chair:**

Prof. Anne-Catherine Bachoud-Lévi (MD, PhD), National Centre of reference for Huntington's disease, Henri Mondor Hospital, AP-HP, Creteil, France

email: bachoud@gmail.com

Phone: +336 80 72 84 17

**• Steering Committee**

- Prof. Jean-Marc Burgunder (MD), Swiss HD Center, NeuroZentrumSiloah and Department of Neurology, University of Bern, Bern, Switzerland

- Prof. Monica Busse (PhD), Centre for Trials Research, Cardiff University, United Kingdom

- David Craufurd (MB.BS, MSc, FRCPsych), Manchester Centre for Genomic Medicine, Division of Evolution and Genomic Sciences, School of Biological Sciences, Faculty of Biology, Medicine and Health, University of Manchester; and Saint Mary's Hospital, Manchester University NHS Foundation Trust, Manchester Academic Health Science Centre, Manchester, United Kingdom

- Prof. Joaquim Ferreira (MD, PhD), Clinical Pharmacology Unit, Instituto de Medicina Molecular, Lisbon, Portugal

- Prof. Giuseppe De Michele (MD), Department of Neurosciences, Federico II University, Naples, Italy

- Daniela Rae (MSc), Department of Clinical Genetics, NHS Grampian, Aberdeen, Scotland, United Kingdom

- Ralf Reilmann (MD, PhD), George-Huntington-Institute & Department of Radiology, Universitaetsklinikum Muenster, & Department of Neurodegenerative Diseases and Hertie-Institute for Clinical Brain Research, University of Tuebingen, Germany

- Prof. Anne Rosser (MA, PhD, MB.BChir, FRCP), IPMCN, School of Medicine, Cardiff University, United Kingdom

- Prof. Klaus Seppi (MD), Department of Neurology, Medical University Innsbruck, Innsbruck, Austria

- Ferdinando Squitieri (MD), Huntington and Rare Diseases Unit, IRCCS Casa Sollievo della Sofferenza, San Giovanni Rotondo, Italy

- Katia Youssov (MD), National Centre of reference for Huntington's disease, Henri Mondor Hospital, AP-HP, Creteil, France

**• French National Committee**

- Prof. Anne-Catherine Bachoud-Lévi (MD, PhD), National Centre of reference for Huntington's disease, Henri Mondor Hospital, AP-HP, Creteil, France

- Prof. Christophe Verny (MD, PhD), Centre National de Référence des Maladies Neurodégénératives Service de Neurologie and UMR CNRS 6214 INSERM U1083, CHU d'Angers, Angers, France.

- Katia Youssov (MD), National Centre of reference for Huntington's disease, Henri Mondor Hospital, AP-HP, Creteil, France

- Charles Perrine (MD, PhD), National Center of reference for Huntington's disease, Genetic department, Sapêtrière Hospital, Paris, France.

- Clarisse Scherer-Gagou (MD), Centre Hospitalier Universitaire d'Angers, Département de Neurologie, Angers, France

- Olivier Audrey (MSc), Département de Neurologie, Centre Hospitalier Universitaire d'Angers, Angers, France

**• United Kingdom National Committee**

- Daniela Rae (MSc), Department of Clinical Genetics, NHS Grampian, Aberdeen, Scotland, United Kingdom

- David Craufurd (MB.BS, MSc, FRCPsych), St Mary's Hospital, Central Manchester University Hospitals NHS Foundation Trust, Manchester Academic Health Science Centre, Manchester, United Kingdom

**• Project Manager**

- Renaud Massart (PhD), National Centre of reference for Huntington's disease, Henri Mondor Hospital, AP-HP, Creteil, France
