# Supplementary material for: International Guidelines for the Treatment of Huntington's Disease
Source: Front Neurol. 2019 Jul 3;10:710. doi: 10.3389/fneur.2019.00710 (PMC6618900; doi:10.3389/fneur.2019.00710)
Supplement: Supplementary file 2 [file Data_Sheet_1.PDF]

Appendix 2.

Recommendations and Symptoms

Given that any symptoms of Huntington's disease (HD) may be worsened by stress, fatigue and intercurrent disorders (e.g. anxiety, digestive disorders, infectious or painful conditions, etc.), these aspects must be assessed and should be treated with appropriate measures in parallel with treating the Huntington's symptoms (Professional agreement).

Motor disorders

The wide spectrum of motor manifestations are the best known and the most visible symptom in Huntington's disease. Among them, involuntary movements (i.e. chorea) are the most obvious. However, while the diagnosis of manifest HD is based on the presence of motor symptoms, these are frequently preceded by cognitive and behavioral symptoms (1). While motor symptoms are easily detected, and might be the source of anxiety and ostracism, they are often well tolerated by the patients and their proxies in contrast to cognitive and behavioral symptoms that often lead to family and social/professional's issues.

Chorea

Chorea is characterized by abnormal, involuntary, spontaneous, uncontrollable, irregular, intermittent, non-rhythmic and aimless movements affecting the trunk, the face and the limbs. They are worsened by fatigue and challenges such as emotional stress, and usually disappear during sleep. Affecting about 90% of the patients, chorea is a key element of establishing the clinical diagnosis, but is not always a marker of the severity of the disease (2). Chorea may be absent at the beginning of HD or appear transiently during the course of the disease, and is rarely present with juvenile onset. After motor onset, chorea usually progresses towards a peak phase and then commonly decreases in severity in the later stages of HD, at which time it may disappear alongside an increase in rigidity and/or dystonia (3).

Recommendations

1. Drug treatment should be considered if chorea causes the patient distress or discomfort (Professional agreement).

2. Tetrabenazine was shown to have a beneficial effect on chorea (Grade A) (4–20) and is one of the first-line treatments for this symptom.

3. Tetrabenazine has to be used with caution due to the potential of adverse events, which should be explained to patients and relatives/caregivers (Professional agreement).

4. Tetrabenazine is contraindicated when the patient suffers from not well-managed depression or suicidal thoughts, as specified in the product monographs.

5. Tiapride (21–27), sulpiride (28–33) (Grade B), olanzapine (34–43), risperidone (28,44–49), pimozide (9,24,50,51) and aripiprazole (52–57) (Grade C) showed efficacy on chorea and are first-line treatments for this symptom in particular when the patients have associated personality and/or behavioural or psychotic disorders.

6. Haloperidol has a national marketing authorization for chorea in some countries (Grade C) (58–63). It should be considered as second-line treatment but should be used with caution due to the risk of major apathy (Professional agreement).

7. Because of contradictory results in various trials, amantadine cannot be recommended to treat chorea (Grade B) (63–67). However, despite the poor tolerability (confusion and legs oedema) of amantadine several experts of the multidisciplinary group, consulted to evaluate the recommendations, reported a beneficial effect of amantadine to treat chorea.

8. On a regular basis, ongoing treatments should be re-evaluated for their efficacy and tolerability. If effective and well-tolerated, changing ongoing treatment is not recommended (Professional agreement).

9. It may be necessary to reduce the dose or change a neuroleptic or tetrabenazine if the patient experiences adverse events such as worsening of apathy or extrapyramidal side effects (Professional agreement).

10. Monotherapy to treat chorea is preferred because combination therapy (e.g. tetrabenazine and neuroleptic) increases the risk of adverse effects and may complicate the management of non-motor symptoms. Adjunct therapy should therefore be considered on a case-by-case basis for patients who do not respond significantly to monotherapy (Professional agreement).

11. Riluzole (Grade A) (39,68–71) and memantine (Grade C) (44,72–74) are not indicated for the management of chorea.

12. Globus pallidus Deep Brain Stimulation (DBS) (either internal or external) is not yet recommended in severe pharmacoresistant chorea (Grade C) (75–86). The technic is currently assessed in therapeutic trials.

13. In the presence of disturbing chorea, appropriate protective measures (for meal time, washing, bedding, transfers...) should be put in place in order to avoid traumatic injury or chokes. Rehabilitation specialists such as occupational therapists, psychomotor therapists, speech therapists, physiotherapists can help identify appropriate assistive technology devices and positioning techniques (Professional agreement).

14. Where there are recurrent oral-lingual injuries due to biting, mouth guards (splints) may be prescribed, in collaboration with a dentist/oral health specialist where possible. The choking risk due to mouth guards should also be considered (Professional agreement).

15. As an adjuvant measure, therapies that might act by relaxing the patient (e.g. aquatherapy or bathing) might be of benefit to improve transitorily chorea symptoms (Professional agreement).

Dystonia

Dystonia is highly prevalent in HD patients (90%) and is characterized by abnormal postures that may affect all body segments and is frequently associated with rigidity (54–59)(87). When

dystonia manifests in HD, the intensity varies from a slight intermittent abnormal posture, without any impact on independence, to severe twitch of muscles with major impact on movements and functions of daily living. It can lead to impaired chewing and swallowing (for dystonia of the face and the neck), joint deformities, and compromise a comfortable sitting position and prevent secure ambulation (especially when severe axial dystonia is present).

Recommendations
1. Both active rehabilitation, with patient participation, and passive rehabilitation, without voluntary participation of patients, in physiotherapy are recommended as a preventive measure in order to maintain the range of joint motion, limit postural and musculoskeletal deformities and, prevent the development of contractures (Professional agreement).

2. The injection of botulinum toxin in the case of focal dystonia (e.g. cervical dystonia, blepharospasm, oromandibular dystonia) or to prevent secondary deformities (e.g. flexum, equinus) should be performed by a trained professional (Professional agreement).

3. The concomitant use of anticholinergic agents to prevent the side effects of neuroleptics has not demonstrated efficacy and should not be used (Professional agreement).

4. The use of adapted equipment, in particular customised chairs, can provide an optimised and comfortable environment in view of the dystonia-related deformities (Professional agreement).

Rigidity
Rigidity is an increase in muscle tone leading to a resistance to passive movement that can induce joint stiffness and limited range of motion. In HD, it might be related to spasticity and/or parkinsonism (with bradykinesia).
Bradykinesia is the slowness of initiation of voluntary movement with a reduction of speed and amplitude, particularly of repetitive actions. *Akinesia* is a severe degree of bradykinesia leading to inability to initiate voluntary movement. In HD, bradykinesia frequently coexists with involuntary choreatic movements. At late stages, when choreatic activity declines, most HD patients develop an akinetic-rigid syndrome often with a generalized increased tone. Juvenile or late-onset patients often exhibit a predominantly bradykinetic phenotype with or without rigidity but little to no chorea (88). This phenotype might be misdiagnosed as atypical parkinsonism or a psychiatric condition.

Recommendations
Only low levels of scientific evidence have been provided for the treatments of rigidity and because of the risk of adverse events, they should be used with caution. Nevertheless, rigidity symptoms might be distressing for patients and the following treatments might be attempted on a case by case basis (Professional agreement).

1. Rigidity may be increased or induced by the use of neuroleptics or tetrabenazine. If this impacts the functional capacity of the patient, a reduction in dosage or the withdrawal of neuroleptics and/or tetrabenazine should be considered taking into account overall benefit on chorea and/or behavioural symptoms vs. severity of rigidity (Professional agreement).

2. Levodopa may provide partial and temporary relief of the akinetic–rigid symptoms of HD, especially in juvenile forms (Grade C) (89–94). Treatment with levodopa should be started gradually and the total daily dose is usually lower than in Parkinson's disease (Professional agreement).

3. A case report suggested that amantadine may be used (Grade C) (95), no consensus has been reached among members to recommend its use to treat rigidity.

4. Case reports suggested that dopamine agonists may be used (Grade C) (96,97), no consensus has been reached among members to recommend their use to treat rigidity.

5. Physiotherapy is recommended to improve or maintain mobility and prevent the development of contractures and joint deformity (Grade C) (98).

Akathisia
Akathisia is a syndrome characterized by unpleasant sensations of "inner" restlessness that manifests as an inability to sit still. It can coexist with chorea.

Recommendations
1. In case of akathisia, an iatrogenic cause should be investigated as the priority (Professional agreement).

2. Tetrabenazine (Grade C) (11,13) and haloperidol may cause akathisia in HD. Neuroleptics and occasionally SSRIs may also cause akathisia as specified in the product monographs.

3. In case of akathisia, reducing the dose or changing the treatment may be helpful. Olanzapine, risperidone and aripiprazole should be considered (Professional agreement).

Swallowing disorders
Swallowing disorders can occur in patients at the early stages of the disease and become a major problem in later stages due to the accumulation of multiple factors: eating too fast because of impulsive food intake, inadequate mastication, tendency to go for another bite before swallowing the last one, premature involuntary swallowing, deficits of tongue movements, tendency to keep leftover food in the mouth after swallowing (99). These disorders induce repeated choking and can lead to secondary bronchopulmonary infections or even cardiac arrest. The frequency of these disorders are not known.

Recommendations
1. Regular assessment of swallowing disorders should be provided throughout the progression of the disease (Grade C) (100).

2. When patients start experiencing swallowing disorders, referral to a Speech and Language Therapist/ Speech pathologist is recommended as good practice (Grade C) (100–102).

3. Ancillary assessments that may help in managing swallowing disorders in HD include: generalised motor skills (co-ordination, posture, tone), respiratory status, dental health,

mood, behaviour and emotional status, cognition, nutrition and hydration status (Professional agreement).

4. Provision of information and advice, on food textures and consistency and food modifications, bolus size and placement, safe swallowing procedures, elimination of distractions and on focusing attention on just one task at a time can help to avoid aspirations and leads to improvement of swallowing disorders (Professional agreement).

5. Oral-facial exercise with swallow sequence individualization and cough post swallow may improve swallowing difficulties (Professional agreement).

6. Advice on posture and positional changes after a multi-disciplinary review might help to develop an individualized management plan (Professional agreement).

7. Videofluoroscopy assessment in some cases can help to visualize the whole swallowing process and to identify the most appropriate compensatory techniques adapted to each patient (Grade C) (101).

8. The education of family and carers is important, especially as the disease progresses as they are often managing the eating, drinking and swallowing regime (Professional agreement).

9. For severe swallowing disorders impacting nutrition and quality of life of the patient, the use of a gastrostomy device (Percutanous Endoscopic Gastrostomy (PEG)) may be considered and should be discussed on a case-by-case basis with the patient and the caregivers (Professional agreement).

10. When PEG is used, the halting of any form of oral nutrition is not necessarily required. When possible and desired by the patient, some forms of oral nutrition may be preserved to maintain pleasure and socialization associated with meals (Professional agreement).

11. PEG should be anticipated and discussed with relatives and patients still able to understand the benefits and burdens of the methods (Professional agreement).

12. Before advanced stages of the disease, patients should be educated to make an informed choice concerning the PEG methods even if they can change their decision at any time (Professional agreement).

13. In some cases, treating chorea might help in improving swallowing problems (Professional agreement). However, side effects of treatments for chorea (e.g. sedation, attention, parkinsonism) might also negatively impact swallowing capacities (Professional agreement).

14. Olanzapine has been reported to improve swallowing disorders (Grade C) (34).

Myoclonus

Myoclonus refers to sudden muscle contractions, brief and involuntary, axial, in extremities or generalized, similar to spams and jerks in epileptic seizures but not related epilepsy. In HD, myoclonus can be observed in a predominant akineto-rigid phenotype and can be associated with an at rest or action tremor, especially in the juvenile forms but also in later-onset forms. The prevalence of this symptom is estimated at less than 2% (103). In juvenile forms, non-epileptic myoclonus can coexist with epilepsy.

Recommendations

1. In case of myoclonus impacting the functional capacity of the patients, associated or not with clinical epileptic seizures, treatment with sodium valproate or clonazepam, used alone or in combination, and in escalating doses, is recommended (Grade C) (103–110).

2. Levetiracetam is a therapeutic alternative for the same indication (Professional agreement).

3. In case of myoclonus of cortical origin (attested by electroencephalography) that is not associated with epileptic seizures, piracetam has a marketing authorization, but the necessary dose is over 12g per day (Grade C) (107).

4. Benzodiazepines, in particular clonazepam, may be used to manage myoclonus whilst remaining vigilant with regard to adverse effects such as somnolence and increasing falls, and the risk of drug-dependence (Professional agreement).

Gait and balance disorders

Gait and balance disorders impairments include disruption of cadence regulation, increased variability of step width and length, disturbed initiation and increased postural sway (111). These develop as a result of the progressive complex movement disorder seen in HD adding to the overall burden of motor morbidity with falls and loss of independence in HD (112).

Recommendations

1. Generally, interventions for gait and balance should start as early as possible and be continued and adapted throughout the progression of the disease (Grade C) (111,113–116).

2. Rehabilitative methods (e.g. physiotherapy and occupational therapy) may improve walking and balance disorders (Grade B) (117–129) and prevent from their main complications (falls, fractures, loss of autonomy) (Professional agreement).

3. Falls can be reduced through the introduction of falls prevention programs, gait, core stability and balance training facilitated by a physiotherapist (home or hospital program) (Grade C) (115,118,119,125).

4. Attention training is also suggested to improve gait and walking (Professional agreement).

5. Pharmaceutical management of chorea may improve walking and balance as they can be affected by chorea (Grade C) (29,37,38,56).

6. The adverse effects of tetrabenazine and neuroleptics (drowsiness, parkinsonism, bradykinesia, dystonia) may also aggravate walking disorders, which means that they should always be used cautiously and regularly reassessed (Professional agreement).

7. Maintaining physical activity (for example Yoga, relaxation, balneotherapy, Tai Chi Chuan, Qi Gong, gentle gymnastics and dance) is encouraged and may improve walking and balance (Professional agreement).

8. Taking part in properly supervised, low impact exercise and walking is recommended (Professional agreement).

9. The use of a four-wheeled walker should be considered to improve stability and reduce fall risk (Grade B) (130).

10. The use of assistive devices and aids (wheelchair) and/or protection (helmet or knee, elbow and wrist protections) should be considered to prevent complications from falls (Professional agreement).

11. A Physiotherapist or Occupational Therapist should guide an assessment for the appropriate use of assistive devices (Professional agreement).

Bruxism

Bruxism is an involuntary clenching with excessive contraction of the jaw muscles. It typically causes lateral movements (or front to back) responsible for gnashing and can lead to tooth damage. It can be nocturnal, diurnal or both. The cause of bruxism in HD is not clear but it may be related to dystonia and/or chorea of the jaw. Bruxism prevalence in HD is low (around 2%) (131).

Recommendations

1. Injecting botulin toxin A into the masseter muscles is proposed as the first-line treatment. Repeating the injections every 3 to 6 months may be needed if the bruxism reappears (Grade C) (132,133).

2. Customized protective mouth guards may be used to reduce the complications of bruxism on a case-by-case basis, mostly in early stage patients (Professional agreement).

3. For now, no oral pharmacological treatment can be recommended for bruxism symptom (Professional agreement).

4. Bruxism may occur as a side effect of neuroleptics, in particular haloperidol (Grade C) (132,133), thus reducing their dose should be considered (Professional agreement).

5. Bruxism may occur as a side effect of serotonin reuptake inhibitors and thus reducing the dose should be considered (Professional agreement).

Manual dexterity

Manual dexterity can be impaired secondary to chorea/dystonia/akinesia/rigidity but also occur in their absence – due to abnormal motor planning and sequencing. Impairments of hand fine motor control has been objectified in the coordination of grip forces (134) and finger tapping regularity (135).

Recommendations

1. No pharmacological agent is recommended for the treatment of manual dexterity specifically (Professional agreement).

2. Olanzapine (37,38) and tetrabenazine (16) may possibly have a beneficial effect on dexterity as a result of reducing chorea (Grade C).

3. Neuroleptics and tetrabenazine may have a detrimental effect on dexterity by aggravating other symptoms such as bradykinesia (Professional agreement).

4. Management with physiotherapy and occupational therapy may be useful to reduce the functional impact of fine motor skill deterioration (Grade B) (128).

5. The occupational therapist may suggest adaptive aids to compensate for the deterioration of manual dexterity (adapted cutlery, computer keyboard, adapted telephone, etc.) (Professional agreement).

Global motor capacities

Recommendations

1. Early referral to a physiotherapist is recommended in order to facilitate the development of a therapeutic relationship, promote sustainable exercise behaviours and ensure long-term functional independence (Professional agreement).

2. Physiotherapy and/or exercise programs are beneficial for the overall functional ability, motor function and independence in HD, in combination with pharmacological treatments (Grade B) (115,117–127,129) .

3. Exercise programs should be personalized (considering abilities and exercise capacity), goal directed and task specific (Grade B) (122).

4. Cell therapy yield contradictory results and is not a treatment of HD but at an experimental stage (Professional agreement).

Cognitive disorders

Cognitive deficits appear frequently before motor symptoms (1). However, by convention "disease onset" is taken to be the onset of the motor symptoms. They are, in addition to behavioral symptoms, the major cause of family disruption and social withdrawal (136). Cognitive symptoms cause intense psychological discomfort and a sense of powerlessness that can lead to behavioral symptoms.

Recommendations

1. Based on present knowledge, no pharmacological treatment is recommended for the treatment of cognitive symptoms. Latrepirdine (137,138), Riluzole (68,69,71), Coenzyme Q10 (139,140) (Grade A), Donezepil (141,142), Rivagstimine (143–146) (Grade B) were not found to be efficacious in treating cognitive problems.

2. Multiple rehabilitation strategies (speech therapy, occupational therapy, cognitive and psychomotricity) might improve or stabilise transitorily cognitive functions (executive functions, memory, language...) at some point of time in the course of the disease (Grade B) (147).

Executive functions and attention
Executive functions refer to the functions that allow the realization of complex task in daily living. They consist in a set of functions mostly dedicated to cognitive and behavior control and adaptation: act inhibition (self-regulation of impulsivity), errors control (inhibition), sustained and divided attention in concomitant activities, working memory, flexibility (capacity to shift from a task to another one or changing perspective), adapting to circumstances, planning activities or sequences, as well as initiation and motivation. High demanding activities are included in this set of functions: reasoning and problem solving. All these capacities may be impaired in HD, even at the premanifest stages (prevalence in pre-HD: 9% and in early-HD: 44%) (148), and thus impose adaptation from the environment, organization support including proactivity in planning appointments, behavior or daily life activities like cooking.

Recommendations
1. It is better to help the patients organize themselves and initiate activities (drawing up lists, a diary, schedule and external incentives) rather than substitute for them, as long as they do not endanger themselves. This approach might help them maintain their independence for as long as possible (Professional agreement).

2. Treatment for anxiety and depression may help to improve executive function and in particular problems with concentration, attention and memory (Professional agreement).

3. Sedative drugs (e.g. benzodiazepines), neuroleptics and tetrabenazine indicated for another symptom of HD should be closely monitored as they have the potential to impair executive functions and attention. (Professional agreement).

4. Cognitive stimulation through rehabilitation (speech therapy, occupational therapy, neuropsychology, psycho-motor therapy) may improve aspects of executive functioning, such as planning and initiation (Grade C) (149).

5. Driving ability correlates with global cognitive performance and total functional capacity (Grade C) (150). When driving capacities are questioned, direct assessment by appropriate professionals, according to the country, is recommended (Professional agreement).

Bradyphrenia

Bradyphrenia is defined by slowing of cognitive information processing and a prolongation of reaction time depending on the complexity of the cognitive task (151). It does not refer to any specific cognitive function and becomes more apparent with HD disease progression.

Recommendations
1. Management is based on giving the patient enough time to process information and perform a task and avoiding time-pressured situations (Professional agreement).

2. Cognitive stimulation as part of rehabilitation (speech therapy, occupational therapy, neuropsychology and psycho-motor therapy) may be beneficial (Professional agreement).

Language and communication disorders
So–called *language disorders* can be divided in speech and language disorders *per se*. Speech disorders consist of slurred and slowed speech causing dysarthria, inappropriate pauses or bursts of speech, and progressive reduction in verbal fluency (152). Regarding language, syntax and morphology (conjugation and affixation) impairments appears early in the disease course, with progressive difficulties in understanding and producing complex sentences. Reduction of lexical capacities appears later. This often goes unnoticed and may cause misunderstanding and impaired communication with proxies and professionals.

Recommendations
1. Speech and Language Therapists can play a major role in assessing and managing communication problems in HD at all stages of the disease. Early referral is recommended (Grade C) (153).

2. The changing communication needs of the person with HD will be monitored and reassessed throughout the course of the disease to plan effective management strategies at all stages (Grade C) (153).

3. Assessment of language should include: orofacial movements, respiratory function in speech, breath control and coordination, phonation, articulation, intelligibility, comprehension and communication abilities (Grade C) (153,154).

4. The communication disorder in HD is variable, requires comprehensive assessment of language and of other factors such as mood, motivation and behaviour (Professional agreement).

5. Communication strategies and techniques may include: management options (e.g. voice therapy techniques) and advice on facilitation of communication (e.g. allowing time for communication, reduction of environmental distractions and noise) (Professional agreement).

6. Communication may be improved by simple technics (e.g. gestures, repeating and rephrasing and use of yes/no questions) or tools (e.g. pen, paper, letter boards, phones) (Professional agreement).

7. People with HD generally prefer to attempt verbal communication as long as possible, even when speech becomes hard to understand. Family members and other communication

partners should be educated on how to support and encourage this (Professional agreement).

8. Augmentative and alternative communication (talking mats) can compensate for communication difficulties in HD and can increase the individual’s chance of participation in daily life. These strategies need to be implemented whilst there is still motivation and a capacity to learn (Grade C) (155).

9. Multi-disciplinary input such as Speech & Language Therapy and Physiotherapy help to retain communication and social interaction (Professional agreement).

Social cognition

Social cognition impairments refer to a set of symptoms that affect relationships and social behavior. The most studied are the inability to recognize emotion others (156) but also to express emotions, both through facial expression or through the voice. Patients are often described as “selfish” and “self-centered” by their proxies but scientific characterization of this is lacking. Executive function impairments can make difficult for the patients, who cannot communicate correctly (stressed by language disorders), to express their feelings. Theory of mind capacities, namely the capacity to infer other thoughts or feeling, are also reported to be impaired in patients (156). Furthermore, motor impairments can create a "facial mask", often misunderstood as indifference.

Recommendations

1. Improvement of behavioural disorders (e.g. with antidepressants or neuroleptics depending on the associated symptomatology) may help with social and family integration (Professional agreement).

2. Impact of SSRI or neuroleptics on social interaction *per se* has not yet been properly assessed to allow any recommendation specific to this domain.

3. Explaining the patients’ disorders to their family, health care professionals or to their colleagues may facilitate the patient's social relationships (Professional agreement).

4. Third party intervention (care giver, education assistant, nurse, nursing auxiliary, social worker, healthcare coordinator) may stimulate patients' social interaction (Professional agreement).

Memory

Memory disorders are frequently reported in HD and may be confounded with or exacerbated by attention disorders. They are mostly characterized by difficulties in learning new information, and retrieving information acquired, but not in the storage of information (unlike Alzheimer's disease) (157).

Recommendations

1. Strategies such as establishing and keeping a regular daily routine, organising a schedule, keeping a diary and drawing up a "to do" list may compensate memory loss (Professional agreement).

2. Rehabilitative approaches (speech therapy or neuropsychology) may help memory as part of an overall intervention plan (stimulation of executive function, rehabilitating the working memory, language work, learning recovery strategies) (Professional agreement).

3. Specifically, domain-specific transcoding (verbal and visual) may help in recalling items (Professional agreement).

4. Sedative drugs (e.g. benzodiazepines) and neuroleptics or tetrabenazine may impact negatively on memory (Professional agreement).

Disorientation

Disorientation, both in time and space, appear during the progression of HD but temporal orientation is altered earlier (158–161). Patients commonly have planning difficulties and shifts in the sleeping and/or wake phases, but they are rarely disorientated in space, except at advanced stages.

Recommendations

1. Investigations should be carried out to detect any potential intercurrent cause for a confusional state (Professional agreement).

2. Establishing a regular routine and milestones enables the patient to manage their time better. This routine should be in tune with the patient's environment as much as possible (Professional agreement).

3. For some patients, external stimuli (reminders, alarms) may be useful and help them to organise their time (Professional agreement).

Visual spatial and visual perceptual disorders

Visuospatial and visual perceptual disorders appear late in the course of the disease through interference with the integration and understanding of visual information (161).

Recommendations

1. It may be useful to make the patient's environment safe (padding furniture) to minimise falls and shocks linked to visual spatial difficulties and aggravated by motor disorders (walking and balance disorders, chorea) (Professional agreement).

Psychiatric disorders

Behavioral symptoms may appear before the motor diagnosis of the disease. They are, in addition to and in conjunction with with cognitive symptoms, the major cause of family disruption, social isolation, and withdrawal.

Recommendations

1. Management should be based on the identification of the underlying triggers causing changes in mood or behaviour. Patients should be given the opportunity to express their worries and frustrations (Professional agreement).

2. Using methods to calm and reassure patients is a major component of care of psychiatric disorders (Professional agreement).

3. Based on data from other neurodegenerative conditions, mindfulness-based cognitive therapy and Acceptance and Commitment Therapy may be useful in HD (Professional agreement).

Depression

Depression (low mood) is one of the most common psychiatric symptoms seen in HD populations from 32 % in pre-HD to 65% in HD (148,162) and may affect patients at any stage of the disease, even before motor manifestation (163). It is important because it has a significant negative impact on quality of life. Thus, vigilance to detect and treat depression is required at all stages of the disease. Depression can sometimes be difficult to recognize in patients whose ability to express themselves verbally is impaired.

Recommendations

1. An antidepressant may be suggested if depression occurs in HD (Grade B) (164–169). The choice of antidepressant is based on potential drug interactions and tolerance.

2. It is recommended to use a selective serotonin reuptake inhibitor (SSRI) or a serotonin noradrenaline reuptake inhibitor (SNRI), or alternatively Mianserin or Mirtazapine, in case of sleep disruption (Professional agreement).

3. The duration of treatment is generally for over 6 months and can be for several years (Professional agreement).

4. The effectiveness of the prescribed antidepressant should be assessed. In absence of efficacy, the dosage should be modified or an alternative antidepressant might be proposed (Professional agreement).

5. If depression is thought to be an adverse effect of other medication, then, rather than sudden discontinuation, the dosage of the responsible drug (e.g. tetrabenazine or an anti-psychotic) should be reduced gradually (Professional agreement).

6. Psychotherapy and cognitive behavioural therapy should be considered when patients are able to participate in them, enabling earlier detection of mood changes (Professional agreement).

7. In case of recurrent depression, long-term mood-stabiliser treatment may be introduced in complement to the treatment of the current episode to prevent relapses (Professional agreement).

8. In the case of resistant depression, or depression associated with psychotic symptoms a psychiatrist should be consulted (Professional agreement).

9. In case of severe depression associated or not with psychotic symptoms and resistant to oral medications, electroconvulsive therapy (ECT) may be suggested under the guidance of a psychiatrist (Grade C) (170–172).

Suicide

Suicidal ideation or attempts are common in HD (173) and correlate with family history of suicide, a history of previous suicide attempts and the presence of depression, especially in prodromal stages (174). Impulsive suicide, outside of a depressive identified context, has also been reported (175). The prevalence of suicide ideation is highly variable regarding the way it was assessed. In cohort follow-up in Europe it reaches 8.8% (162) and in psychiatric interviews 20% (175).

Recommendations

1. Suicide risk should be assessed in Huntington's disease irrespective of the stage, being particularly vigilant at the time of diagnosis (symptomatic or presymptomatic) and when the disease starts to impact on day-to-day life (Professional agreement).

2. Prevention of suicide includes treating risk factors such as underlying depression, social isolation and impulsivity (Professional agreement).

Irritability/aggressivness

Irritability is a very common symptom in HD and is reported in 38 to 73 % of patients (148,162,176). This disorder is of fluctuating nature, characterized by impatience and a tendency to become angry in response to minimal provocation. Overflow and loss of control are favored by impulsivity, and can lead to aggressive behavior towards self or others, and, rarely, to criminal behavior. This symptom can be caused by the frustrations felt by the patient because of the great loss of his capacities, and by troubles in expressing himself, as well as by neurological/psychological fatigue brought by the latter.

Recommendations

1. Before initiating pharmacological treatment, possible environmental causes for the patient's frustration and irritability should be explored (Professional agreement).

2. In order to reduce irritability, behavioural strategies should be considered. A structured plan with a regular routine in a calming environment is desirable. In addition, psycho-education for the patient's family regarding diversion strategies (such as changing the subject or suggesting a new activity) should be attempted, in order to avoid confrontation as much as possible (Professional agreement).

3. Whilst the majority of experts recommend using an SSRI as a first line for irritability (Grade C) (177,178), it may be necessary to use them at or near the maximum recommended dose in order to be effective. A substantial minority of experts would favour instead low dose antipsychotics.

4. Irritable patients who do not benefit from an SSRI alone at the maximum recommended doses may benefit from combination therapy with Mianserine or Mirtazapine, especially when sleep disorders are present (note of caution – Mianserine and Mirtazapine can worsen irritability and insomnia).

5. In patients with aggressive behaviour (e.g. threatening or assaulting people), the recommended first-line treatment is a neuroleptic (Grade C) (47,179,180).

6. To treat irritability, olanzapine, risperidone and tiapride are preferred over haloperidol, which has poorer tolerability (Professional agreement).

7. When using neuroleptics, monotherapy should be encouraged as far as possible before any combination is envisaged (Professional agreement).

8. For non-compliant patients, injectable olanzapine and risperidone are a possibility and are preferred over sustained-release haloperidol (Professional agreement).

9. Aripiprazole is a second line neuroleptic which can be useful for treating irritability, but caution is required because in some cases agitation or increased irritability may be observed (Professional agreement).

10. In case of overt aggression associated with depression, neuroleptic treatment should be associated with sedative antidepressants (e.g. mirtazapine or mianserine) (Professional agreement).

11. If irritability does not respond to antidepressant and/or neuroleptics, a mood stabiliser such as valproic acid, lamotrigine (Grade C) (181,182), lithium, valpromide or carbamazepine (Professional agreement) can be added.

Apathy

Apathy has been defined by Levy and Czernecki (183) as 'a quantifiable reduction in goal-directed behavior', manifesting clinically as a reduction in interest, spontaneity, motivation and drive. In patients with HD it is compounded by emotional blunting, resulting in social withdrawal and lack of concern for others. It is the most frequent psychological and behavioral symptom in HD, especially in the middle and later stages, causing a severe reduction in the activities of daily living and often being a source of conflict in the family.
Apathy prevalence increases with disease progression (162).

With regard to cognitive and psychological symptoms, apathy and irritability are the two faces of the same coin (184). A patient can be apathic the morning and irritable the afternoon, depending on the situation. As for irritability, apathy can be caused by environmental and psychological issues. Apathy may also be an adaptive response when the patient feels overwhelmed by too much stimulation (HD patients are more sensitive to noise and environmental interferences), or with the feeling that his/her disease is progressing.

Recommendations

1. It is important to explain the various aspects and causes of the apathy to the family circle (Professional agreement).

2. Some sedative medication (e.g. benzodiazepine, neuroleptics and tetrabenazine) may increase apathy, thus avoiding unnecessary prescription or reduce dosage is recommended (Professional agreement).

3. It is necessary to look for depression, which may increase apathy even if it is difficult to disentangle the two symptoms. If depression is suspected, an SSRI should be tried (Professional agreement).

4. Personalised cognitive stimulation, establishing routines and a structured programme of activities is recommended when possible (Professional agreement).

5. A professional intervention at home can improve compliance and reduce the patient's opposition and irritability (Professional agreement).

Anxiety

Anxiety as defined by the uncomfortable feeling of nervousness or worry about something that is happening or might happen in the future, is common in HD. The prevalence of anxiety in manifest HD ranged from 13% to 71% (185). It appears to be associated with depression, suicide, irritability, quality of life, pain, illness beliefs, and coping. Anxiety does not increase with disease progression.

Anxiety is linked to the other symptoms (motor and cognitive), as the patient is anxious because of the loss of essential functions, and correlated to family, social and economic issues, and to the burden of his pathology (and the one of his proxies – as HD is a family disorder).

Recommendations

1. If the anxiety requires pharmacological treatment, an SSRI or SNRI are first line treatments, especially when associated with depression (Professional agreement).

2. On-demand prescription of an anxiolytic (a benzodiazepine or buspirone) might be beneficial, but caution is required because of the associated risk of worsening or causing falls (Professional agreement).

3. Olanzapine (34), risperidone (186) (Grade C) or quetiapine (Professional agreement) are valuable therapeutic alternatives in the treatment of anxiety when other treatments fail.

4. Cyamemazine and pregabalin have a marketing authorisation in some countries for anxiety and may be useful in HD (Professional agreement).

Obsessive-compulsions behaviors and perseverations

Obsessions are defined by recurrent and persistent thoughts, ideas or images that do not let the mind rest, causing anxiety. True obsessions, according to this definition, are not very common in patients with HD, but *perseveration* is very common, particularly in the middle and later stages (176). Perseveration may be defined as the repetition of a thought, behavior or emotion beyond the psychological context in which it arose, and in patients with HD these repetitive thoughts and behaviors can persist for hours, months, or even years after the original trigger. Patients have little or no insight into the problem (in contrast to obsessional thoughts, which are distressing and recognized as abnormal); however, it has been shown that perseveration is the one behavioral symptom in HD which has a significant negative impact on the quality of life of family members and caregivers (187).

Recommendations
1. Over the course of HD, symptoms may change and repetitive thoughts may replace obsessive–compulsive disorder (Professional agreement).

2. The distinction between obsessive–compulsive phenomena and perseverations is important for the care strategy, both requiring differential approaches (Professional agreement).

Perseverations - recommendations
1. It is important to help those around the patients to understand that the perseverations are a symptom of HD (Professional agreement).

2. If pharmacological treatment is necessary for perseverative symptoms, an SSRI could be prescribed (Grade C) (188), in particular when symptoms are associated with anxiety (Professional agreement).

3. It is often necessary to use SSRIs at or close to the manufacturer's maximum recommended dose levels (Professional agreement).

4. There are two valuable therapeutics for ideational perseverations: olanzapine (34) and risperidone (47) (Grade C), in particular when they are associated with irritability (Professional agreement).

5. Psychological therapies such as cognitive behaviour therapy (CBT) are probably ineffective for perseverations (Professional agreement).

True obsessive-compulsive disorders - recommendations
1. True obsessive–compulsive phenomena are sensitive to psychological intervention, such as Cognitive Behavioural Therapy, as the first-line treatment in non-cognitively impaired patients (Professional agreement).

2. If pharmacological treatment is necessary for obsessive-compulsive phenomena, a SSRI should be prescribed as first-line treatment (Grade C) (188).

Impulsivity
Impulsivity is often reported in HD, together with irritability and aggressive behavior (40%) (189). It consists of acting without prior planning, which can lead to unpredictable behavior. When impulsivity is associated with depression or irritability, there is a significant increased risk of self-harm or suicide or aggressiveness.
Impulsivity may be the result of cognitive impairments, which lead to an intense frustration towards patience, the patient being in the incapacity to wait or to deal cognitively with planning. The language difficulties lead to the inability to express correctly, so the patient cannot explain what stresses him/her. Impulsivity may then be an adaptive response.

Recommendations

1. When impulsivity is associated with depression or personality disorders, there is a risk of auto- or hetero-aggressiveness, which justifies the prescription of a neuroleptic in combination with a SSRI (Professional agreement).

2. Olanzapine, risperidone and aripiprazole are first line treatments for impulsivity and haloperidol is second line treatment because of its poorer tolerability profile (Professional agreement).

3. Long-term mood-stabiliser treatment (e.g. valproate, carbamazepine, lithium) may be introduced in the case of mood lability and impulsivity (Professional agreement). Note that the use of valproate should strongly be avoided in women of childbearing capacity not using efficient contraceptive method.

Sexual disorders
Sexual disorder. Up to 85% of men and 75% of women in HD experience sexual disorders (190). Decreased libido is the most common symptom while hypersexuality or disinhibited behavior are rarer, but can cause significant problems in relationships. Repetitive hypersexual behaviors are often a result of perseveration. Scarce cases of paraphilia are reported.

Recommendations
1. Identifying the existence of sexual disorders and determining their triggers and their impact on relationships is important (Professional agreement).

2. In the case of decreased libido, an iatrogenic cause should be investigated (for example, the use of an SSRI). Reducing the dose or substituting the treatment responsible may also be suggested (Professional agreement).

3. In the case of decreased libido (when both members of the couple regard the situation as a problem), psychological support and/or referral to a specialist in psychosexual disorders might be useful (Professional agreement).

4. In the case of erectile dysfunction, treatment for impotence may be suggested and seeking the opinion of an endocrinologist and/or a specialist in psycho-sexual disorders may be useful (Professional agreement).

5. In case of impotence, prescription of phophoesterase 5 inhibitors (e.g. sildenafil) should be considered in the clinic when asked for by the patient and his sexual partner (Professional agreement).

6. A behavioural and psychological approach is useful in the case of hyper sexuality, by re-establishing appropriate standards of behaviour in the patient's social setting (Professional agreement).

7. If hypersexuality involves social discomfort or violence, the proposed first-line treatment is a neuroleptic (Grade C) (61) and/or a SSRI, especially if the sexual problems are part of a broader issue of behavioural disorders (Professional agreement).

8. In case of hypersexuality, some thymoregulators (e.g. carbamazepine, lamotrigine) may be attempted (Professional agreement).

9. If the treatment for hypersexuality with neuroleptics and/or SSRI is not successful, the addition of or substitution for an anti-androgen may be proposed (Grade C) (191–193) under the guidance of a specialist in sexual disorders or an endocrinologist (Professional agreement).

10. Where hypersexuality poses a risk to others, specific measures should immediately be put in place (referral to a psychiatrist, alerting, isolation, etc.) (Professional agreement).

Hallucinations/delusions

Hallucinations are defined as a perception without an object, at which the subject adheres to and reacts as if the perception came from outside. Their frequency is low in HD : 2 to 3% in clinical cohorts (189,194), which may be underestimated since in later stages psychotic symptoms are more prevalent.

Delusions, with a frequency around 11% in clinical cohorts (194), are false beliefs based on incorrect inferences about external reality, the cultural and social context to which the patient belongs.

Recommendations

1. The use by the patient of psychotropic agents should be searched for and interrupted in case of hallucinations and delusions (Professional agreement).

2. Neuroleptics are the first line treatment for hallucinations and delusions (Grade C) (34,43,44,47,49,52,53,57,180,195–200).

3. It is recommended to prescribe olanzapine (34,43), risperidone (44,47,49,197) or quetiapine (195,199), less likely to induce extrapyramidal side effects than first generation neuroleptics (Grade C).

4. Clozapine (Grade C) (49,54,180,198,200) and first-generation neuroleptics (Professional agreement) may be suggested as second-line treatment.

5. Clozapine should be proposed as the first-line treatment in the case of akinetic forms of HD with debilitating parkinsonian symptoms (Professional agreement).

6. For non-compliant patients, sustained-release injectable neuroleptics may be considered (Professional agreement).

7. If pharmacological treatments fail, the option of ECT can be discussed with psychiatrists (Grade C) (170,172,201).

8. Psychiatric intervention and support are particularly useful in the case of psychotic disorders occurring in HD, for treatment adjustments (Professional agreement).

9. Perseverative ideation can sometimes mimic psychotic symptoms, and in such circumstances the patient may benefit from treatment with serotoninergic antidepressants (e.g. SSRIs, mirtazapine or clomipramine) in combination with an atypical neuroleptic (e.g. Olanzapine or risperidone) (Professional agreement).

Agitation

Recommendations

1. In case of agitation, priority should be given to identifying environmental or somatic triggers (bladder distension, fecal impaction, pain, etc.) in order to treat the underlying cause, especially in the advanced stages of the disease when communication difficulties exist (Professional agreement).

2. When agitation is associated with an anxiety disorder, a benzodiazepine should be prescribed as needed to reduce the risk of dependence and falls (Professional agreement).

3. Some benzodiazepines (e.g. midazolam) may be useful in emergency situations (Professional agreement).

4. Long-term treatment with benzodiazepines (e.g. clonazepam and diazepam) should be avoided as much as possible but remains necessary in some patients (Professional agreement).

5. In the case of extreme agitation, and if there are associated behavioural and personality disorders, it is advised to prescribe a neuroleptic, and ideally one which has intramuscular administration (Grade C) (61,181,191,195,202,203).

Other disorders

Other symptoms than motor, cognitive and psychiatric disorders are often present. Among those, weight loss, dysphagia and sleep disturbance are not unfrequently the most prominent symptoms. As they may cause discomfort, they should be looked for in order to limit them when present.

Sleep-related difficulties

Sleep disorders are common in HD. Around two-thirds of HD patients suffer from sleep disorders, with diverse causes such as depression, anxiety, intrinsic alteration in the circadian sleep-wake rhythm, and involuntary movements during sleep inducing awakenings (204,205). They may present as difficulties in falling asleep and/or early awakenings in the middle of the night followed by insomnia. They may be associated with aimless wandering, and lead to difficulties in coping by the proxies. However, disturbances of diurnal rhythm (day-night reversal, etc.) are probably more common than simple insomnia in HD patients.

Recommendations

1. Potential underlying cause of sleep-related difficulties (e.g. depressive syndrome, anxiety, severe involuntary movements) should be investigated (Professional agreement).

2. Simple lifestyle and dietary strategies, avoiding long nap, having no stimulants after 4pm and getting up at a set time are recommended as the first-line treatment of insomnia (Professional agreement).

3. When lifestyle strategies are ineffective to treat insomnia, prescribing a hypnotic may be suggested, preferably at the lowest possible dosage and for a short duration, to avoid the risk of drug dependence (Professional agreement).

4. Some agents may be proposed in place of a hypnotic and for a long duration (e.g. mianserin, mirtazapine and antihistaminic drugs) as they have a reduced tendency for causing dependency (Professional agreement).

5. Melatonin may be suggested in case of sleep phase inversion (Professional agreement).

6. A neuroleptic (e.g. olanzapine) should be prescribed in the evening when sleep disorders are associated with behavioural disorders or chorea (Professional agreement).

Incontinence

Urinary incontinence may either be multifunctional or linked to a deterioration of the frontal lobe control centers, causing an overactive bladder with urge incontinence and/or unannounced urination (206).

Recommendations

1. Where there is urinary incontinence, a precipitating factor should always be investigated (urinary infection, prostate disease, particularly in men over the age of 50) (Professional agreement).

2. It is useful to investigate the presence of diurnal unexpected complete urination (complete and sudden bladder emptying, without urge) for which carbamazepine at a dose of 200mg may be of benefit (Grade C) (206).

3. Nocturnal urinary incontinence combined with diurnal unexpected urination may also be improved by taking carbamazepine at a dose of 200mg/day with a dose-related effect (Grade C) (206).

4. In the case of an overactive bladder with leakage and urge incontinence, antimuscarinic therapy may be tried (Professional agreement).

5. The most selective antimuscarinics (e.g. trospium, solifenacin, mirabegron) should be given over the less selective (oxybutynin) whilst watching out for the appearance of potential side effects, in particular confusional state (Professional agreement).

6. If, after few weeks, the incontinence therapy has not been effective, it should be stopped (Professional agreement).

7. If simple therapeutic measures have failed, it is advised to undergo urodynamic testing to help guide the choice of drug therapy and to consult a urologist if necessary (Professional agreement).

8. In all cases, it is recommended to implement simple lifestyle strategies: urination before every outing and at regular times (with external stimulation if the patient has cognitive difficulties) (Professional agreement).

Pain

Pain assessment is sometimes difficult because of communication disorders. Moreover, because of communication's disorders and a tendency for these patients not to complain, pain is often related to non-verbal language and behavioral disorders such as irritability and restfulness.

Recommendations

1. Behavioural change or worsening of involuntary movements should trigger the search for an underlying source of discomfort, and in particular pain (Professional agreement).

Dental diseases/dental pain

Dental pain. Patients suffer from poor oral health for a variety of reasons, including impaired motor ability (e.g. difficulties brushing teeth) or reduced motivation to maintain oral health, the use of drugs affecting salivary secretion, frequent dental trauma due to falls and injuries, bruxism and possible (as yet unproven) deleterious effect of the mutation on the dental condition.

Recommendations

1. Multidisciplinary teamwork, especially with dietitians to avoid highly cariogenic foods, is recommended (Grade C) (207,208).

2. Verbal and written instructions on how to provide good oral hygiene at home should be given to patients and carers (Grade C) (208,209).

3. It is essential to promote good oral hygiene, in particular the use of a fluoride rich toothpaste and oral hygiene aids (collis-curve toothbrush, enlarged or weighted handles, mouth angle expanders, electric tooth brush) (Professional agreement).

4. Dental care including descaling by a dentist or dental hygienist should be carried out at least once a year but should be more frequent in the later stages of the disease (3-4 times per year) (Professional agreement).

5. At later stages of the disease, treatment options should be discussed carefully and in advance (Professional agreement).

6. Restorative treatment should be high quality and low maintenance (Professional agreement).

7. It is essential to retain functional teeth for as long as possible and dentures should be considered carefully, assessing the risk of aspiration (Professional agreement).

8. If dentures are fitted, they should be radio-opaque labelled (Professional agreement)

9. Treatment intervention, especially in late stage disease may require conscious sedation (midazolam, Diazepam) or general anaesthesia in a hospital setting (Grade C) (209–211).

Digestive disorders

Recommendations
1. In view of the frequency of digestive disorders in HD (e.g. constipation, diarrhea, vomiting) and their impact on the quality of life of patients, routine assessment for these symptoms is recommended in order to ensure their management (Professional agreement).

2. The diagnostic workup of digestive disorders in HD is comparable to the overall population and should be conducted by the relevant specialists (general and digestive examination, biological and radiological tests, scan, fibroscopy, colonoscopy, etc.) (Professional agreement).

3. There is no symptomatic treatment of digestive disorders specific to Huntington's patients. (Professional agreement)

4. Faecal impaction should be routinely investigated where there is constipation/ diarrhoea ("false" diarrhoea) and/or vomiting (Professional agreement).

5. Vomiting is sometimes intractable. If no specific aetiology is identified, the following should be considered: staggering meals, reviewing the patients' posture during and after the meal, and possibly reducing antichoreic agents, in particular neuroleptics (Professional agreement).

Perspiration
Perspiration. Excessive sweating can occur at all stages of HD. It can be associated with other autonomic disorders and reflects discomfort or emotional burst when sudden.

Recommendations
1. In the case of excessive perspiration, care must be taken to ensure patients are well hydrated, monitored and that their fluid and electrolyte balance is adjusted (Professional agreement).

2. Extra clothes or covers should be removed in case of excessive perspiration (Professional agreement).

3. Thyroid function and the possibility of infection should be assessed in case of excessive perspiration (Professional agreement).

Weight loss

Weight loss is often present in HD, sometimes prior to the appearance of other symptoms. Weight loss might occur despite normal, or even high calorie intake, due to a significant energy expenditure in HD patients. It can also be caused by swallowing disorders, depressive syndrome with reduced appetite or gastrointestinal disturbance and gut abnormalities due to enteric neuron dysfunction (212). High baseline Body Mass Index are associated with slower decline of the condition independent of mutant CAG repeat size suggesting a systemic metabolism etiology (213).

Recommendations
1. Good nutritional care is a fundamental element of the management of HD (Grade C) (214,215).

2. Due to the variability in energy requirements and the potential for rapid weight loss, early assessment by a dietitian or nutritionist is recommended (Professional agreement).

3. Regular timely reviews of nutritional needs are useful throughout the progression of the disease (Professional agreement).

4. When considering weight loss, other factors such as swallowing ability, cognitive changes, behaviour, mood and general functional ability should be considered to determine possible other causes of weight loss (Grade C) (101,141,215–217).

5. A multi-disciplinary approach is recommended and may include a Speech Language Therapist and an Occupational Therapist to assist with swallowing (see swallowing disorder), positioning and feeding aids (Professional agreement).

6. Screening tools for malnutrition (e.g. MUST (malnutrition Universal Screening Tool) are recommended. Screening should be repeated according to clinical need (Professional agreement).

7. A high BMI within normal values should be maintained if possible (Professional agreement).

8. Medical and/or social intervention is recommended when unintended weight loss is higher than 10% within last 3-6 month (Professional agreement).

9. Medical and/or social intervention is recommended when BMI is less than 20kg/m^2 and unintentional weight loss of 5% is observed within last 3-6 month (Professional agreement).

10. When weight loss is observed, high-calorie and high-protein food supplements should be prescribed under instruction and monitored by a dietician/nutritionist (Grade C) (218–220).

11. Individuals with HD have higher energy requirements and the calorific intake necessary to control weight loss can sometimes be as high as 5,000 kcal/day, especially during the advanced stages of the disease (Professional agreement).

12. A Mediterranean diet may improve Quality of Life and nutritional composition (Grade C) (221).

13. In case of the initiation of antidepressant and/or neuroleptic treatments, treatments inducing weight gain should be preferred in patients with significant weight loss, whilst treatments inducing weight loss should be avoided (these effects can vary from one patient to another) (Grade C) (222).

14. Advanced care planning is essential and alternative feeding methods (PEG) should be anticipated and discussed with relatives and patients still able to understand the benefits and risks of the intervention (Professional agreement).

15. Before advanced stages of the disease, patients should be educated to make an informed choice concerning the PEG methods even if they can change their decision later (Professional agreement).

16. The benefits and risks of enteral feeding should be discussed and recorded in medical records or advanced directives as per patient wishes. This should ideally not be done in a crisis situation (Professional agreement).

17. If the patient lacks capacity, a discussion between professionals and families is essential. Decisions should be made on a case by-case basis in line with local and national policies (Professional agreement).

Hypersalivation

Hypersalivation. Excessive drooling can be troublesome in HD patients when associated with a salivary incontinence (caused by poor oral occlusion and or fault swallowing).

Recommendations

1. In the absence of a specific treatment for HD, drugs used in other chronic diseases may be considered to reduce salivary secretion: scopolamine given percutaneously, atropine given orally (used orally in eye drops for example) or other drugs that have an anticholinergic effect (amitriptyline), whilst watching out for iatrogenic risks, in particular confusional state, constipation, ocular hypertension and urinary retention (Professional agreement).

2. Injections of botulinum toxin into the salivary glands may be considered in a specialised setting if oral or oral mucosa treatment options have not induced benefit or were not well tolerated (Professional agreement).

Lung functions

Reduced lung function and respiratory muscle strength are not only associated with end stage disease but occur much earlier, with evidence of some upper airway changes in presymptomatic individuals and reduction of cough effectiveness, reduced lung volume, and impaired respiratory strength by mid-disease. Along with changes in posture reduced exercise capacity, these impairments negatively impact respiratory function, leaving patients vulnerable to respiratory infections.

Recommendations

1. Home-based respiratory muscle training program appeared to improve pulmonary function in manifest Huntington's disease patients but had only a small effect on swallowing function, dyspnea and exercise capacity (Grade B) (223).

References

1. Paulsen JS, Miller AC, Hayes T, Shaw E. Cognitive and behavioral changes in Huntington disease before diagnosis. Handb Clin Neurol. 2017;144:69-91.
2. Mahant N, McCusker EA, Byth K, Graham S, Huntington Study Group. Huntington's disease: clinical correlates of disability and progression. Neurology. 28 oct 2003;61(8):1085-92.
3. Shoulson I. Huntington's disease. In: Diseases of the nervous system Clinical neurobiology. Saunders (W.B.) Co Ltd; 1986. p. 1258-67.
4. Gilligan BS, Wodak J, Veale JL, Munro OR. Tetrabenazine in the treatment of extrapyramidal dyskinesias. Med J Aust. 4 nov 1972;2(19):1054-6.
5. Astin KJ, Gumpert EW. Letter: Tetrabenazine in chorea. Lancet. 23 mars 1974;1(7856):512.
6. Dorsey R, Biglan K, Eberly S, Auinger P, Brocht A, Umeh CC, et al. Use of Tetrabenazine in Huntington Disease Patients on Antidepressants or with Advanced Disease: Results from the TETRA-HD Study. PLoS Curr. 13 nov 2011;3:RRN1283.
7. Fasano A, Cadeddu F, Guidubaldi A, Piano C, Soleti F, Zinzi P, et al. The long-term effect of tetrabenazine in the management of Huntington disease. Clin Neuropharmacol. déc 2008;31(6):313-8.
8. Ferrara JM, Mostile G, Hunter C, Adam OR, Jankovic J. Effect of tetrabenazine on motor function in patients with huntington disease. Neurol Ther. déc 2012;1(1):5.
9. Fog R, Pakkenberg H. Combined nitoman-pimozide treatment of Huntington's chorea and other hyperkinetic syndromes. Acta Neurol Scand. 1970;46(2):249-51.
10. Frank S, Ondo W, Fahn S, Hunter C, Oakes D, Plumb S, et al. A study of chorea after tetrabenazine withdrawal in patients with Huntington disease. Clin Neuropharmacol. juin 2008;31(3):127-33.
11. Frank S. Tetrabenazine as anti-chorea therapy in Huntington disease: an open-label continuation study. Huntington Study Group/TETRA-HD Investigators. BMC Neurol. 18 déc 2009;9:62.
12. Huntington Study Group. Tetrabenazine as antichorea therapy in Huntington disease: a randomized controlled trial. Neurology. 14 févr 2006;66(3):366-72.
13. Huang CY, McLeod JG, Holland RT, Elliot C. Tetrabenazine in the treatment of Huntington's chorea. Med J Aust. 17 avr 1976;1(16):583-4.
14. Kingston D. Tetrabenazine for involuntary movement disorders. Med J Aust. 30 juin 1979;1(13):628-30.
15. McArthur AW, Pollock M, Smidt NA. Combined therapy with tetrabenazine and pimozide in Huntington's chorea: pilot study. N Z Med J. 25 févr 1976;83(558):114-6.
16. McLellan DL, Chalmers RJ, Johnson RH. A double-blind trial of tetrabenazine, thiopropazate, and placebo in patients with chorea. Lancet. 26 janv 1974;1(7848):104-7.
17. Ondo WG, Tintner R, Thomas M, Jankovic J. Tetrabenazine treatment for Huntington's disease-associated chorea. Clin Neuropharmacol. déc 2002;25(6):300-2.
18. Pakkenberg H. The effect of tetrabenazine in some hyperkinetic syndromes. Acta Neurol Scand. 1968;44(3):391-3.

19. Scott LJ. Tetrabenazine: for chorea associated with Huntington's disease. CNS Drugs. 1 déc 2011;25(12):1073-85.
20. Soutar CA. Tetrabenazine for Huntington's chorea. Br Med J. 3 oct 1970;4(5726):55.
21. Chouza C, Romero S, Lorenzo J, Camano JL, Fontana AP, Alterwain P, et al. [Clinical trial of tiapride in patients with dyskinesia (author's transl)]. Sem Hop. 25 mars 1982;58(12):725-33.
22. Csanda E, Tarczy M, Jelencsik I. Tiapride treatment of abnormal movements and painful conditions. Vol. 60. 1984. 3006 p.
23. Deroover J, Baro F, Bourguignon RP, Smets P. Tiapride versus placebo: a double-blind comparative study in the management of Huntington's chorea. Curr Med Res Opin. 1984;9(5):329-38.
24. Girotti F, Carella F, Scigliano G, Grassi MP, Soliveri P, Giovannini P, et al. Effect of neuroleptic treatment on involuntary movements and motor performances in Huntington's disease. J Neurol Neurosurg Psychiatry. août 1984;47(8):848-52.
25. Kocher R, Hobi V. [Treatment of dyskinesias with tiapride (author's transl)]. Sem Hop. 18 déc 1980;56(47-68):2033-4.
26. Roos RA, Buruma OJ, Bruyn GW, Kemp B, van der Velde EA. Tiapride in the treatment of Huntington's chorea. Acta Neurol Scand. janv 1982;65(1):45-50.
27. Zenglein JP, Baldauf E, Hussaini M. [A new treatment of Huntington's chorea. Report of three cases (author's transl)]. Sem Hop. 18 sept 1978;54(25-28):871-4.
28. Reveley MA, Dursun SM, Andrews H. A comparative trial use of sulpiride and risperidone in Huntington's disease: a pilot study. J Psychopharmacol (Oxford). janv 1996;10(2):162-5.
29. Knowling MR, Wrench W. Treatment of Huntington's chorea with sulpiride. S Afr Med J. 2 févr 1991;79(3):169.
30. Petit H, Havet JP. [Huntington's chorea and sulpiride]. Lille Med. sept 1971;16(7):1019-21.
31. Quinn N, Marsden CD. A double blind trial of sulpiride in Huntington's disease and tardive dyskinesia. J Neurol Neurosurg Psychiatry. août 1984;47(8):844-7.
32. Ranouil R. [Value of dogmatil in the treatment of Huntington's disease. Apropos of a psychiatric and statistical survey in Haute-Vienne]. Therapeutique. juin 1973;49(5):413-4.
33. Reveley MA, Dursun SM, Andrews H. Improvement of abnormal saccadic eye movements in Huntington's disease by sulpiride: a case study. J Psychopharmacol (Oxford). janv 1994;8(4):262-5.
34. Squitieri F, Cannella M, Porcellini A, Brusa L, Simonelli M, Ruggieri S. Short-term effects of olanzapine in Huntington disease. Neuropsychiatry Neuropsychol Behav Neurol. janv 2001;14(1):69-72.
35. Benazzi F. Rapid onset of tardive dyskinesia in Huntington disease with olanzapine. J Clin Psychopharmacol. août 2002;22(4):438-9.
36. Bogelman G, Hirschmann S, Modai I. Olanzapine and Huntington's disease. J Clin Psychopharmacol. avr 2001;21(2):245-6.
37. Bonelli RM, Mahnert FA, Niederwieser G. Olanzapine for Huntington's disease: an open label study. Clin Neuropharmacol. oct 2002;25(5):263-5.
38. Bonelli RM, Niederwieser G, Tribl GG, Költringer P. High-dose olanzapine in Huntington's disease. Int Clin Psychopharmacol. mars 2002;17(2):91-3.
39. Bonelli RM, Niederwieser G, Diez J, Költringer P. Riluzole and olanzapine in Huntington's disease. Eur J Neurol. mars 2002;9(2):183-4.
40. Dipple HC. The use of olanzapine for movement disorder in Huntington's disease: a first case report. J Neurol Neurosurg Psychiatry. juill 1999;67(1):123-4.

41. Etchebehere EC, Lima MC, Passos W, Maciel Júnior JA, Santos AO, Ramos CD, et al. Brain SPECT imaging in Huntington's disease before and after therapy with olanzapine. Case report. Arq Neuropsiquiatr. sept 1999;57(3B):863-6.
42. Laks J, Rocha M, Capitão C, Domingues RC, Ladeia G, Lima M, et al. Functional and motor response to low dose olanzapine in Huntington's disease: case report. Arq Neuropsiquiatr. déc 2004;62(4):1092-4.
43. Paleacu D, Anca M, Giladi N. Olanzapine in Huntington's disease. Acta Neurol Scand. juin 2002;105(6):441-4.
44. Cankurtaran ES, Ozalp E, Soygur H, Cakir A. Clinical experience with risperidone and memantine in the treatment of Huntington's disease. J Natl Med Assoc. août 2006;98(8):1353-5.
45. Dallocchio C, Buffa C, Tinelli C, Mazzarello P. Effectiveness of risperidone in Huntington chorea patients. J Clin Psychopharmacol. févr 1999;19(1):101-3.
46. Erdemoglu AK, Boratav C. Risperidone in chorea and psychosis of Huntington's disease. Eur J Neurol. mars 2002;9(2):182-3.
47. Johnston TG. Risperidone long-acting injection and Huntington's disease: case series with significant psychiatric and behavioural symptoms. Int Clin Psychopharmacol. mars 2011;26(2):114-9.
48. Meco G, Bonifati V, Alessandri A, Brusa L. Letters. Human Psychopharmacology: Clinical and Experimental. 1 juill 1995;10(4):353-4.
49. Parsa MA, Szigethy E, Voci JM, Meltzer HY. Risperidone in treatment of choreoathetosis of Huntington's disease. J Clin Psychopharmacol. avr 1997;17(2):134-5.
50. Arena R, Iudice A, Virgili P, Moretti P, Menchetti G. Huntington's disease: clinical effects of a short-term treatment with pimozide. Adv Biochem Psychopharmacol. 1980;24:573-5.
51. Lal S, De la Vega C, Garelis E, Sourkes TL. Apomorphine, pimozide, L-Dopa and the probenecid test in Huntington's chorea. Psychiatr Neurol Neurochir. avr 1973;76(2):113-7.
52. Yavuz KF, Ulusoy S, Alnıak İ. Aripiprazole treatment for choreoathetoid and psychotic symptoms of Huntington's disease. J Neuropsychiatry Clin Neurosci. 2013;25(2):E31.
53. Oulis P, Mourikis I, Konstantakopoulos G, Papageorgiou SG, Kouzoupis AV. Aripiprazole in the treatment of olanzapine-resistant psychotic and motor symptoms of Huntington's disease. J Neuropsychiatry Clin Neurosci. 2010;22(3):352c.e4-352.e5.
54. Edlinger M, Seppi K, Fleischhacker W, Hofer A. Treatment of psychotic and behavioral symptoms with clozapine, aripiprazole, and reboxetine in a patient with Huntington's disease. Int Clin Psychopharmacol. juill 2013;28(4):214-6.
55. Brusa L, Orlacchio A, Moschella V, Iani C, Bernardi G, Mercuri NB. Treatment of the symptoms of Huntington's disease: preliminary results comparing aripiprazole and tetrabenazine. Mov Disord. 15 janv 2009;24(1):126-9.
56. Ciammola A, Sassone J, Colciago C, Mencacci NE, Poletti B, Ciarmiello A, et al. Aripiprazole in the treatment of Huntington's disease: a case series. Neuropsychiatr Dis Treat. 2009;5:1-4.
57. Lin W-C, Chou Y-H. Aripiprazole effects on psychosis and chorea in a patient with Huntington's disease. Am J Psychiatry. sept 2008;165(9):1207-8.
58. Barr AN, Fischer JH, Koller WC, Spunt AL, Singhal A. Serum haloperidol concentration and choreiform movements in Huntington's disease. Neurology. janv 1988;38(1):84-8.

59. Caraceni T, Calderini G, Consolazione A, Riva E, Algeri S, Girotti F, et al. Biochemical aspects of Huntington's chorea. J Neurol Neurosurg Psychiatry. juin 1977;40(6):581-7.
60. Janati A. Kluver-Bucy syndrome in Huntington's chorea. J Nerv Ment Dis. oct 1985;173(10):632-5.
61. Jhanjee A, Anand KS, Bajaj BK. Hypersexual features in Huntington's disease. Singapore Med J. juin 2011;52(6):e131-133.
62. Koller WC, Trimble J. The gait abnormality of Huntington's disease. Neurology. oct 1985;35(10):1450-4.
63. Saran BM, Klein MS, Benay EM. Clinical evaluation of amantadine and haloperidol in Huntington's chorea. J Clin Psychiatry. juin 1980;41(6):221.
64. Heckmann JM, Legg P, Sklar D, Fine J, Bryer A, Kies B. IV amantadine improves chorea in Huntington's disease: an acute randomized, controlled study. Neurology. 10 août 2004;63(3):597-8; author reply 597-598.
65. Lucetti C, Del Dotto P, Gambaccini G, Dell' Agnello G, Bernardini S, Rossi G, et al. IV amantadine improves chorea in Huntington's disease: an acute randomized, controlled study. Neurology. 24 juin 2003;60(12):1995-7.
66. O'Suilleabhain P, Dewey RB. A randomized trial of amantadine in Huntington disease. Arch Neurol. juill 2003;60(7):996-8.
67. Verhagen Metman L, Morris MJ, Farmer C, Gillespie M, Mosby K, Wuu J, et al. Huntington's disease: a randomized, controlled trial using the NMDA-antagonist amantadine. Neurology. 10 sept 2002;59(5):694-9.
68. Huntington Study Group. Dosage effects of riluzole in Huntington's disease: a multicenter placebo-controlled study. Neurology. 9 déc 2003;61(11):1551-6.
69. Landwehrmeyer GB, Dubois B, de Yébenes JG, Kremer B, Gaus W, Kraus PH, et al. Riluzole in Huntington's disease: a 3-year, randomized controlled study. Ann Neurol. sept 2007;62(3):262-72.
70. Rosas HD, Koroshetz WJ, Jenkins BG, Chen YI, Hayden DL, Beal MF, et al. Riluzole therapy in Huntington's disease (HD). Mov Disord. mars 1999;14(2):326-30.
71. Seppi K, Mueller J, Bodner T, Brandauer E, Benke T, Weirich-Schwaiger H, et al. Riluzole in Huntington's disease (HD): an open label study with one year follow up. J Neurol. oct 2001;248(10):866-9.
72. Beister A, Kraus P, Kuhn W, Dose M, Weindl A, Gerlach M. The N-methyl-D-aspartate antagonist memantine retards progression of Huntington's disease. J Neural Transm Suppl. 2004;(68):117-22.
73. Hjermind LE, Law I, Jønch A, Stokholm J, Nielsen JE. Huntington's disease: effect of memantine on FDG-PET brain metabolism? J Neuropsychiatry Clin Neurosci. 2011;23(2):206-10.
74. Ondo WG, Mejia NI, Hunter CB. A pilot study of the clinical efficacy and safety of memantine for Huntington's disease. Parkinsonism Relat Disord. oct 2007;13(7):453-4.
75. Biolsi B, Cif L, Fertit HE, Robles SG, Coubes P. Long-term follow-up of Huntington disease treated by bilateral deep brain stimulation of the internal globus pallidus. J Neurosurg. juill 2008;109(1):130-2.
76. Fasano A, Mazzone P, Piano C, Quaranta D, Soleti F, Bentivoglio AR. GPi-DBS in Huntington's disease: results on motor function and cognition in a 72-year-old case. Mov Disord. 15 juill 2008;23(9):1289-92.
77. Garcia-Ruiz PJ, Ayerbe J, del Val J, Herranz A. Deep brain stimulation in disabling involuntary vocalization associated with Huntington's disease. Parkinsonism Relat Disord. juill 2012;18(6):803-4.

78. Hebb MO, Garcia R, Gaudet P, Mendez IM. Bilateral stimulation of the globus pallidus internus to treat choreathetosis in Huntington's disease: technical case report. Neurosurgery. févr 2006;58(2):E383; discussion E383.
79. Huys D, Bartsch C, Poppe P, Lenartz D, Huff W, Prütting J, et al. Management and outcome of pallidal deep brain stimulation in severe Huntington's disease. Fortschr Neurol Psychiatr. avr 2013;81(4):202-5.
80. Kang GA, Heath S, Rothlind J, Starr PA. Long-term follow-up of pallidal deep brain stimulation in two cases of Huntington's disease. J Neurol Neurosurg Psychiatry. mars 2011;82(3):272-7.
81. Spielberger S, Hotter A, Wolf E, Eisner W, Müller J, Poewe W, et al. Deep brain stimulation in Huntington's disease: A 4-year follow-up case report. Movement Disorders. 1 mai 2012;27(6):806-7.
82. Zittel S, Moll CKE, Gulberti A, Tadic V, Rasche D, Bäumer T, et al. Pallidal deep brain stimulation in Huntington's disease. Parkinsonism Relat Disord. sept 2015;21(9):1105-8.
83. Gonzalez V, Cif L, Biolsi B, Garcia-Ptacek S, Seychelles A, Sanrey E, et al. Deep brain stimulation for Huntington's disease: long-term results of a prospective open-label study. J Neurosurg. juill 2014;121(1):114-22.
84. Velez-Lago FM, Thompson A, Oyama G, Hardwick A, Sporrer JM, Zeilman P, et al. Differential and better response to deep brain stimulation of chorea compared to dystonia in Huntington's disease. Stereotact Funct Neurosurg. 2013;91(2):129-33.
85. Moro E, Lang AE, Strafella AP, Poon Y-YW, Arango PM, Dagher A, et al. Bilateral globus pallidus stimulation for Huntington's disease. Ann Neurol. août 2004;56(2):290-4.
86. López-Sendón Moreno JL, García-Caldentey J, Regidor I, del Álamo M, García de Yébenes J. A 5-year follow-up of deep brain stimulation in Huntington's disease. Parkinsonism Relat Disord. févr 2014;20(2):260-1.
87. van de Zande NA, Massey TH, McLauchlan D, Pryce Roberts A, Zutt R, Wardle M, et al. Clinical characterization of dystonia in adult patients with Huntington's disease. Eur J Neurol. 2017;24(9):1140-7.
88. Ruocco HH, Lopes-Cendes I, Laurito TL, Li LM, Cendes F. Clinical presentation of juvenile Huntington disease. Arq Neuropsiquiatr. mars 2006;64(1):5-9.
89. Barbeau A. L-dopa and juvenile Huntington's disease. Lancet. 15 nov 1969;2(7629):1066.
90. Jongen PJ, Renier WO, Gabreëls FJ. Seven cases of Huntington's disease in childhood and levodopa induced improvement in the hypokinetic--rigid form. Clin Neurol Neurosurg. 1980;82(4):251-61.
91. Low PA, Allsop JL, Halmagyi GM. Huntington's chorea: the rigid form (Westphal variant) treated with levodopa. Med J Aust. 16 mars 1974;1(11):393-4.
92. Reuter I, Hu MT, Andrews TC, Brooks DJ, Clough C, Chaudhuri KR. Late onset levodopa responsive Huntington's disease with minimal chorea masquerading as Parkinson plus syndrome. J Neurol Neurosurg Psychiatry. févr 2000;68(2):238-41.
93. Vargas AP, Carod-Artal FJ, Bomfim D, Vázquez-Cabrera C, Dantas-Barbosa C. Unusual early-onset Huntingtons disease. J Child Neurol. juin 2003;18(6):429-32.
94. Wang S-C, Lee-Chen G-J, Wang C-K, Chen C-M, Tang L-M, Wu Y-R. Markedly asymmetrical parkinsonism as a leading feature of adult-onset Huntington's disease. Mov Disord. juill 2004;19(7):854-6.
95. Magnet MK, Bonelli RM, Kapfhammer H-P. Amantadine in the akinetic-rigid variant of Huntington's disease. Ann Pharmacother. août 2004;38(7-8):1194-6.

96. Magnet MK, Kapfhammer H-P, Bonelli RM. Cabergoline in Huntington's disease: the first case report. Acta Neurol Scand. mai 2006;113(5):355-6.
97. Bonelli RM, Niederwieser G, Diez J, Gruber A, Költringer P. Pramipexole ameliorates neurologic and psychiatric symptoms in a Westphal variant of Huntington's disease. Clin Neuropharmacol. févr 2002;25(1):58-60.
98. Quinn L, Busse M. Physiotherapy clinical guidelines for Huntington's disease. Neurodegenerative Disease Management. 1 févr 2012;2(1):21-31.
99. Heemskerk A-W, Roos RAC. Dysphagia in Huntington's disease: a review. Dysphagia. mars 2011;26(1):62-6.
100. Monaco AD, Nuzzi A, Parente A, Lavermicocca V, Chiarelli T, Tommaso MD, et al. I03 Swallowing Function In The Early, Middle And Late Stages Of Huntington's Disease. J Neurol Neurosurg Psychiatry. 1 sept 2014;85(Suppl 1):A58-A58.
101. Kagel MC, Leopold NA. Dysphagia in Huntington's disease: a 16-year retrospective. Dysphagia. 1992;7(2):106-14.
102. Giddens C, E. Coleman A, Adams C. Home Program of Speech Therapy in Huntington's Disease. Vol. 18. 2010. 1 p.
103. Saft C, Lauter T, Kraus PH, Przuntek H, Andrich JE. Dose-dependent improvement of myoclonic hyperkinesia due to Valproic acid in eight Huntington's Disease patients: a case series. BMC Neurol. 28 févr 2006;6:11.
104. Carella F, Scaioli V, Ciano C, Binelli S, Oliva D, Girotti F. Adult onset myoclonic Huntington's disease. Mov Disord. avr 1993;8(2):201-5.
105. Novom S, Danna S, Goldberg MA. Intention myoclonus in Huntington's disease. Bull Los Angeles Neurol Soc. avr 1976;41(2):82-4.
106. Previdi P, Borgonovi R. Myoclonus and Huntington's chorea: description of a case. Ital J Neurol Sci. juin 1980;1(3):189-91.
107. Thompson PD, Bhatia KP, Brown P, Davis MB, Pires M, Quinn NP, et al. Cortical myoclonus in Huntington's disease. Mov Disord. nov 1994;9(6):633-41.
108. Vogel CM, Drury I, Terry LC, Young AB. Myoclonus in adult Huntington's disease. Ann Neurol. févr 1991;29(2):213-5.
109. Landau ME, Cannard KR. EEG characteristics in juvenile Huntington's disease: a case report and review of the literature. Epileptic Disord. sept 2003;5(3):145-8.
110. Kereshi S, Schlagenhauff RE, Richardson KS. Myoclonic and major seizures in early adult Huntington's chorea: case-report and electro-clinical findings. Clin Electroencephalogr. avr 1980;11(2):44-7.
111. Rao AK, Muratori L, Louis ED, Moskowitz CB, Marder KS. Spectrum of gait impairments in presymptomatic and symptomatic Huntington's disease. Mov Disord. 15 juin 2008;23(8):1100-7.
112. Delval A, Krystkowiak P, Blatt J-L, Delliaux M, Destée A, Derambure P, et al. [Evolution of locomotion disorders in Huntington's disease]. Neurophysiol Clin. avr 2008;38(2):117-25.
113. Piira A, van Walsem MR, Mikalsen G, Nilsen KH, Knutsen S, Frich JC. Effects of a One Year Intensive Multidisciplinary Rehabilitation Program for Patients with Huntington's Disease: a Prospective Intervention Study. PLoS Curr. 20 sept 2013;5.
114. Piira A, van Walsem MR, Mikalsen G, Øie L, Frich JC, Knutsen S. Effects of a Two-Year Intensive Multidisciplinary Rehabilitation Program for Patients with Huntington's Disease: a Prospective Intervention Study. PLoS Curr. 25 nov 2014;6.
115. Zinzi P, Salmaso D, De Grandis R, Graziani G, Maceroni S, Bentivoglio A, et al. Effects of an intensive rehabilitation programme on patients with Huntington's disease: a pilot study. Clin Rehabil. juill 2007;21(7):603-13.

116. Kegelmeyer D, Fritz N, Kostyk S, Kloos A. J04 The effect of video game-based exercise on dynamic balance and mobility in individuals with Huntington's disease. J Neurol Neurosurg Psychiatry. 1 sept 2010;81(Suppl 1):A40.
117. Thompson JA, Cruickshank TM, Penailillo LE, Lee JW, Newton RU, Barker RA, et al. The effects of multidisciplinary rehabilitation in patients with early-to-middle-stage Huntington's disease: a pilot study. Eur J Neurol. sept 2013;20(9):1325-9.
118. Mirek E, Filip M, Banaszkiewicz K, Rudzińska M, Szymura J, Pasiut S, et al. The effects of physiotherapy with PNF concept on gait and balance of patients with Huntington's disease - pilot study. Neurol Neurochir Pol. 2015;49(6):354-7.
119. Bohlen S, Ekwall C, Hellström K, Vesterlin H, Björnefur M, Wiklund L, et al. Physical therapy in Huntington's disease--toward objective assessments? Eur J Neurol. févr 2013;20(2):389-93.
120. Khalil H, Quinn L, van Deursen R, Dawes H, Playle R, Rosser A, et al. What effect does a structured home-based exercise programme have on people with Huntington's disease? A randomized, controlled pilot study. Clin Rehabil. juill 2013;27(7):646-58.
121. Quinn L, Rao A. Physical Therapy for People with Huntington Disease: Current Perspectives and Case Report. Journal of Neurologic Physical Therapy. 2002;26(3):145.
122. Quinn L, Debono K, Dawes H, Rosser AE, Nemeth AH, Rickards H, et al. Task-specific training in Huntington disease: a randomized controlled feasibility trial. Phys Ther. nov 2014;94(11):1555-68.
123. Busse M, Quinn L, Debono K, Jones K, Collett J, Playle R, et al. A randomized feasibility study of a 12-week community-based exercise program for people with Huntington's disease. J Neurol Phys Ther. déc 2013;37(4):149-58.
124. Busse ME, Wiles CM, Rosser AE. Mobility and falls in people with Huntington's disease. J Neurol Neurosurg Psychiatry. janv 2009;80(1):88-90.
125. Thaut MH, Miltner R, Lange HW, Hurt CP, Hoemberg V. Velocity modulation and rhythmic synchronization of gait in Huntington's disease. Mov Disord. sept 1999;14(5):808-19.
126. Bilney B, Morris ME, Churchyard A, Chiu E, Georgiou-Karistianis N. Evidence for a disorder of locomotor timing in Huntington's disease. Mov Disord. janv 2005;20(1):51-7.
127. Ekwall AIC. Physiotherapy for Patients with Huntington´s Disease : Effects of a Treatment Program with focus on balance and transitions and the Intercorrelation between Assessment Tools [Internet]. 2010 [cité 16 août 2018]. Disponible sur: http://urn.kb.se/resolve?urn=urn:nbn:se:uu:diva-130757
128. Ciancarelli I, Tozzi Ciancarelli MG, Carolei A. Effectiveness of intensive neurorehabilitation in patients with Huntington's disease. Eur J Phys Rehabil Med. avr 2013;49(2):189-95.
129. Delval A, Krystkowiak P, Delliaux M, Dujardin K, Blatt J-L, Destée A, et al. Role of attentional resources on gait performance in Huntington's disease. Mov Disord. 15 avr 2008;23(5):684-9.
130. Kloos AD, Kegelmeyer DA, White SE, Kostyk SK. The impact of different types of assistive devices on gait measures and safety in Huntington's disease. PLoS ONE. 2012;7(2):e30903.
131. Ella B, Ghorayeb I, Burbaud P, Guehl D. Bruxism in Movement Disorders: A Comprehensive Review. J Prosthodont. oct 2017;26(7):599-605.
132. Nash MC, Ferrell RB, Lombardo MA, Williams RB. Treatment of bruxism in Huntington's disease with botulinum toxin. J Neuropsychiatry Clin Neurosci. 2004;16(3):381-2.

133. Tan EK, Jankovic J, Ondo W. Bruxism in Huntington's disease. Mov Disord. janv 2000;15(1):171-3.
134. Reilmann R, Bohlen S, Klopstock T, Bender A, Weindl A, Saemann P, et al. Grasping premanifest Huntington's disease - shaping new endpoints for new trials. Mov Disord. 15 déc 2010;25(16):2858-62.
135. Bechtel N, Scahill RI, Rosas HD, Acharya T, van den Bogaard SJA, Jauffret C, et al. Tapping linked to function and structure in premanifest and symptomatic Huntington disease. Neurology. 14 déc 2010;75(24):2150-60.
136. Ready RE, Mathews M, Leserman A, Paulsen JS. Patient and caregiver quality of life in Huntington's disease. Mov Disord. 15 avr 2008;23(5):721-6.
137. HORIZON Investigators of the Huntington Study Group and European Huntington's Disease Network. A randomized, double-blind, placebo-controlled study of latrepirdine in patients with mild to moderate Huntington disease. JAMA Neurol. janv 2013;70(1):25-33.
138. Kieburtz K, McDermott MP, Voss TS, Corey-Bloom J, Deuel LM, Dorsey ER, et al. A randomized, placebo-controlled trial of latrepirdine in Huntington disease. Arch Neurol. févr 2010;67(2):154-60.
139. Feigin A, Kieburtz K, Como P, Hickey C, Claude K, Abwender D, et al. Assessment of coenzyme Q10 tolerability in Huntington's disease. Mov Disord. mai 1996;11(3):321-3.
140. Huntington Study Group. A randomized, placebo-controlled trial of coenzyme Q10 and remacemide in Huntington's disease. Neurology. 14 août 2001;57(3):397-404.
141. Cubo E, Shannon KM, Tracy D, Jaglin JA, Bernard BA, Wuu J, et al. Effect of donepezil on motor and cognitive function in Huntington disease. Neurology. 10 oct 2006;67(7):1268-71.
142. Fernandez HH, Friedman JH, Grace J, Beason-Hazen S. Donepezil for Huntington's disease. Mov Disord. janv 2000;15(1):173-6.
143. Rot U, Kobal J, Sever A, Pirtosek Z, Mesec A. Rivastigmine in the treatment of Huntington's disease. Eur J Neurol. nov 2002;9(6):689-90.
144. de Tommaso M, Specchio N, Sciruicchio V, Difruscolo O, Specchio LM. Effects of rivastigmine on motor and cognitive impairment in Huntington's disease. Mov Disord. déc 2004;19(12):1516-8.
145. de Tommaso M, Difruscolo O, Sciruicchio V, Specchio N, Livrea P. Two years' follow-up of rivastigmine treatment in Huntington disease. Clin Neuropharmacol. févr 2007;30(1):43-6.
146. Sešok S, Bolle N, Kobal J, Bucik V, Vodušek DB. Cognitive function in early clinical phase huntington disease after rivastigmine treatment. Psychiatr Danub. sept 2014;26(3):239-48.
147. Cruickshank TM, Thompson JA, Domínguez D JF, Reyes AP, Bynevelt M, Georgiou-Karistianis N, et al. The effect of multidisciplinary rehabilitation on brain structure and cognition in Huntington's disease: an exploratory study. Brain Behav. févr 2015;5(2):e00312.
148. Martinez-Horta S, Perez-Perez J, van Duijn E, Fernandez-Bobadilla R, Carceller M, Pagonabarraga J, et al. Neuropsychiatric symptoms are very common in premanifest and early stage Huntington's Disease. Parkinsonism Relat Disord. avr 2016;25:58-64.
149. Metzler-Baddeley C, Cantera J, Coulthard E, Rosser A, Jones DK, Baddeley RJ. Improved Executive Function and Callosal White Matter Microstructure after Rhythm Exercise in Huntington's Disease. J Huntingtons Dis. 2014;3(3):273-83.
150. Devos H, Nieuwboer A, Tant M, De Weerdt W, Vandenberghe W. Determinants of fitness to drive in Huntington disease. Neurology. 6 nov 2012;79(19):1975-82.

151. Papoutsi M, Labuschagne I, Tabrizi SJ, Stout JC. The cognitive burden in Huntington's disease: pathology, phenotype, and mechanisms of compensation. Mov Disord. 15 avr 2014;29(5):673-83.
152. Ludlow CL, Connor NP, Bassich CJ. Speech timing in Parkinson's and Huntington's disease. Brain Lang. nov 1987;32(2):195-214.
153. Saldert C, Fors A, Ströberg S, Hartelius L. Comprehension of complex discourse in different stages of Huntington's disease. Int J Lang Commun Disord. déc 2010;45(6):656-69.
154. Kaploun LR, Saxman JH, Wasserman P, Marder K. Acoustic Analysis of Voice and Speech Characteristics in Presymptomatic Gene Carriers of Huntington's Disease: Biomarkers for Preclinical Sign Onset? Journal of Medical Speech - Language Pathology [Internet]. 1 juin 2011 [cité 20 août 2018]; Disponible sur: https://www.highbeam.com/doc/1G1-260582935.html
155. Ferm U, Sahlin A, Sundin L, Hartelius L. Using Talking Mats to support communication in persons with Huntington's disease. Int J Lang Commun Disord. oct 2010;45(5):523-36.
156. Bora E, Velakoulis D, Walterfang M. Social cognition in Huntington's disease: A meta-analysis. Behav Brain Res. 15 janv 2016;297:131-40.
157. Aretouli E, Brandt J. Episodic memory in dementia: Characteristics of new learning that differentiate Alzheimer's, Huntington's, and Parkinson's diseases. Arch Clin Neuropsychol. août 2010;25(5):396-409.
158. Beste C, Saft C, Andrich J, Müller T, Gold R, Falkenstein M. Time processing in Huntington's disease: a group-control study. PLoS ONE. 5 déc 2007;2(12):e1263.
159. Rao AK, Marder KS, Uddin J, Rakitin BC. Variability in interval production is due to timing-dependent deficits in Huntington's disease. Mov Disord. oct 2014;29(12):1516-22.
160. Cope TE, Grube M, Singh B, Burn DJ, Griffiths TD. The basal ganglia in perceptual timing: timing performance in Multiple System Atrophy and Huntington's disease. Neuropsychologia. janv 2014;52:73-81.
161. Lawrence AD, Watkins LH, Sahakian BJ, Hodges JR, Robbins TW. Visual object and visuospatial cognition in Huntington's disease: implications for information processing in corticostriatal circuits. Brain. juill 2000;123 (Pt 7):1349-64.
162. van Duijn E, Craufurd D, Hubers AAM, Giltay EJ, Bonelli R, Rickards H, et al. Neuropsychiatric symptoms in a European Huntington's disease cohort (REGISTRY). J Neurol Neurosurg Psychiatry. déc 2014;85(12):1411-8.
163. Epping EA, Mills JA, Beglinger LJ, Fiedorowicz JG, Craufurd D, Smith MM, et al. Characterization of depression in prodromal Huntington disease in the neurobiological predictors of HD (PREDICT-HD) study. J Psychiatr Res. oct 2013;47(10):1423-31.
164. Beglinger LJ, Adams WH, Langbehn D, Fiedorowicz JG, Jorge R, Biglan K, et al. Results of the citalopram to enhance cognition in Huntington disease trial. Mov Disord. mars 2014;29(3):401-5.
165. Bonelli RM. Mirtazapine in suicidal Huntington's disease. Ann Pharmacother. mars 2003;37(3):452.
166. Ford MF. Treatment of depression in Huntington's disease with monoamine oxidase inhibitors. Br J Psychiatry. nov 1986;149:654-6.
167. Holl AK, Wilkinson L, Painold A, Holl EM, Bonelli RM. Combating depression in Huntington's disease: effective antidepressive treatment with venlafaxine XR. Int Clin Psychopharmacol. janv 2010;25(1):46-50.
168. Moldawsky RJ. Effect of amoxapine on speech in a patient with Huntington's disease. Am J Psychiatry. janv 1984;141(1):150.

169. Whittier J, Haydu G, Crawford J. Effect of imipramine (Tofranil) on depression and hyperkinesia in Huntington's disease. Am J Psychiatry. juill 1961;118:79.
170. Lewis CF, DeQuardo JR, Tandon R. ECT in genetically confirmed Huntington's disease. J Neuropsychiatry Clin Neurosci. 1996;8(2):209-10.
171. Ranen NG, Peyser CE, Folstein SE. ECT as a treatment for depression in Huntington's disease. J Neuropsychiatry Clin Neurosci. 1994;6(2):154-9.
172. Evans DL, Pedersen CA, Tancer ME. ECT in the Treatment of Organic Psychosis in Huntington's Disease. Convuls Ther. 1987;3(2):145-50.
173. Wesson M, Boileau NR, S Perlmutter J, S Paulsen J, Barton SK, McCormack MK, et al. Suicidal Ideation Assessment in Individuals with Premanifest and Manifest Huntington Disease. J Huntingtons Dis. 27 juill 2018;
174. Fiedorowicz JG, Mills JA, Ruggle A, Langbehn D, Paulsen JS, PREDICT-HD Investigators of the Huntington Study Group. Suicidal behavior in prodromal Huntington disease. Neurodegener Dis. 2011;8(6):483-90.
175. Hubers AAM, van Duijn E, Roos RAC, Craufurd D, Rickards H, Bernhard Landwehrmeyer G, et al. Suicidal ideation in a European Huntington's disease population. J Affect Disord. oct 2013;151(1):248-58.
176. van Duijn E, Kingma EM, van der Mast RC. Psychopathology in verified Huntington's disease gene carriers. J Neuropsychiatry Clin Neurosci. 2007;19(4):441-8.
177. Ranen NG, Lipsey JR, Treisman G, Ross CA. Sertraline in the treatment of severe aggressiveness in Huntington's disease. J Neuropsychiatry Clin Neurosci. 1996;8(3):338-40.
178. De Marchi N, Daniele F, Ragone MA. Fluoxetine in the treatment of Huntington's disease. Psychopharmacology (Berl). 1 janv 2001;153(2):264-6.
179. Désaméricq G, Dolbeau G, Verny C, Charles P, Durr A, Youssov K, et al. Effectiveness of anti-psychotics and related drugs in the Huntington French-speaking group cohort. PLoS ONE. 2014;9(1):e85430.
180. Colosimo C, Cassetta E, Bentivoglio AR, Albanese A. Clozapine in Huntington's disease. Neurology. mai 1995;45(5):1023-4.
181. Grove VE, Quintanilla J, DeVaney GT. Improvement of Huntington's disease with olanzapine and valproate. N Engl J Med. 28 sept 2000;343(13):973-4.
182. Shen Y-C. Lamotrigine in motor and mood symptoms of Huntington's disease. World J Biol Psychiatry. 2008;9(2):147-9.
183. Levy R, Czernecki V. Apathy and the basal ganglia. J Neurol. déc 2006;253 Suppl 7:VII54-61.
184. Bouwens JA, van Duijn E, van der Mast RC, Roos RAC, Giltay EJ. Irritability in a Prospective Cohort of Huntington's Disease Mutation Carriers. J Neuropsychiatry Clin Neurosci. 2015;27(3):206-12.
185. Dale M, van Duijn E. Anxiety in Huntington's Disease. J Neuropsychiatry Clin Neurosci. 2015;27(4):262-71.
186. Duff K, Beglinger LJ, O'Rourke ME, Nopoulos P, Paulson HL, Paulsen JS. Risperidone and the treatment of psychiatric, motor, and cognitive symptoms in Huntington's disease. Ann Clin Psychiatry. mars 2008;20(1):1-3.
187. Read J, Jones R, Owen G, Leavitt BR, Coleman A, Roos RAC, et al. Quality of life in Huntington's disease: a comparative study investigating the impact for those with pre-manifest and early manifest disease, and their partners. J Huntingtons Dis. 2013;2(2):159-75.
188. Patzold T, Brüne M. Obsessive compulsive disorder in huntington disease: a case of isolated obsessions successfully treated with sertraline. Neuropsychiatry Neuropsychol Behav Neurol. sept 2002;15(3):216-9.

189. Craufurd D, Thompson JC, Snowden JS. Behavioral changes in Huntington Disease. Neuropsychiatry Neuropsychol Behav Neurol. déc 2001;14(4):219-26.
190. Reininghaus E, Lackner N. Relationship satisfaction and sexuality in Huntington's disease. Handb Clin Neurol. 2015;130:325-34.
191. Tavares A, Volpe FM. Cyproterone for treatment of hypersexuality in an elderly Huntington's disease patient. Prog Neuropsychopharmacol Biol Psychiatry. 12 déc 2008;32(8):1994-5.
192. Rich SS, Ovsiew F. Leuprolide acetate for exhibitionism in Huntington's disease. Mov Disord. mai 1994;9(3):353-7.
193. Blass DM, Steinberg M, Leroi I, Lyketsos CG. Successful multimodality treatment of severe behavioral disturbance in a patient with advanced Huntington's disease. Am J Psychiatry. déc 2001;158(12):1966-72.
194. Paulsen JS, Ready RE, Hamilton JM, Mega MS, Cummings JL. Neuropsychiatric aspects of Huntington's disease. J Neurol Neurosurg Psychiatry. sept 2001;71(3):310-4.
195. Alpay M, Koroshetz WJ. Quetiapine in the treatment of behavioral disturbances in patients with Huntington's disease. Psychosomatics. févr 2006;47(1):70-2.
196. Madhusoodanan S, Brenner R, Moise D, Sindagi J, Brafman I. Psychiatric and neuropsychological abnormalities in Huntington's disease: a case study. Ann Clin Psychiatry. sept 1998;10(3):117-20.
197. Madhusoodanan S, Brenner R. Use of risperidone in psychosis associated with Huntington's disease. Am J Geriatr Psychiatry. 1998;6(4):347-9.
198. Sajatovic M, Verbanac P, Ramirez LF, Meltzer HY. Clozapine treatment of psychiatric symptoms resistant to neuroleptic treatment in patients with Huntington's chorea. Neurology. janv 1991;41(1):156.
199. Seitz DP, Millson RC. Quetiapine in the management of psychosis secondary to huntington's disease: a case report. Can J Psychiatry. juin 2004;49(6):413.
200. Vallette N, Gosselin O, Kahn JP. [Efficacy of clozapine in the course of Huntington chorea: apropos of a clinical case]. Encephale. avr 2001;27(2):169-71.
201. Nakano T, Ono S, Yamaguchi J, Sugimoto R, Yamaguchi N, Morimoto Y, et al. Modified electroconvulsive therapy for the treatment of refractory schizophrenia-like psychosis associated with Huntington's disease. J Neurol. janv 2013;260(1):312-4.
202. Rej S, Desautels R. Experience with intramuscular zuclopenthixol and medroxyprogesterone acetate in the treatment of agitation and aggression in Huntington's disease. J Neuropsychiatry Clin Neurosci. 2013;25(3):E33-34.
203. Korenyi C, Whittier JR. Drug treatment in 117 cases of Huntington's disease with special reference to fluphenazine (Prolixin). Psychiatr Q. 1967;41(2):203-10.
204. Bellosta Diago E, Pérez Pérez J, Santos Lasaosa S, Viloria Alebesque A, Martínez Horta S, Kulisevsky J, et al. Circadian rhythm and autonomic dysfunction in presymptomatic and early Huntington's disease. Parkinsonism Relat Disord. nov 2017;44:95-100.
205. Arnulf I, Nielsen J, Lohmann E, Schiefer J, Schieffer J, Wild E, et al. Rapid eye movement sleep disturbances in Huntington disease. Arch Neurol. avr 2008;65(4):482-8.
206. Cochen V, Degos JD, Bachoud-Lévi AC. Efficiency of carbamazepine in the treatment of micturitional disturbances in Huntington disease. Neurology. 26 déc 2000;55(12).
207. Lewis D, Fiske J, Dougall A. Access to special care dentistry, part 7. Special care dentistry services: seamless care for people in their middle years--part 1. Br Dent J. 27 sept 2008;205(6):305-17.

208. Manley G, Lane H, Carlsson A, Ahlborg B, Mårtensson Å, Nilsson MB, et al. Guideline for oral healthcare of adults with Huntington's disease. Neurodegenerative Disease Management. 13 janv 2012;2(1):55-65.
209. Boyle CA, Frölander C, Manley G. Providing dental care for patients with Huntington's disease. Dent Update. juin 2008;35(5):333-6.
210. Moline DO, Iglehart DR. Huntington's chorea: review and case report. Gen Dent. avr 1985;33(2):131-3.
211. Cangemi CF, Miller RJ. Huntington's disease: review and anesthetic case management. Anesth Prog. 1998;45(4):150-3.
212. Sciacca S, Favellato M, Madonna M, Metro D, Marano M, Squitieri F. Early enteric neuron dysfunction in mouse and human Huntington disease. Parkinsonism Relat Disord. 2017;34:73-4.
213. van der Burg JMM, Gardiner SL, Ludolph AC, Landwehrmeyer GB, Roos RAC, Aziz NA. Body weight is a robust predictor of clinical progression in Huntington disease. Ann Neurol. sept 2017;82(3):479-83.
214. Brotherton A, Campos L, Rowell A, Zoia V, Simpson SA, Rae D. Nutritional management of individuals with Huntington's disease: nutritional guidelines. Neurodegenerative Disease Management. 13 janv 2012;2(1):33-43.
215. Trejo A, Tarrats RM, Alonso ME, Boll M-C, Ochoa A, Velásquez L. Assessment of the nutrition status of patients with Huntington's disease. Nutrition. févr 2004;20(2):192-6.
216. Leopold NA, Kagel MC. Dysphagia in Huntington's disease. Arch Neurol. janv 1985;42(1):57-60.
217. Nance MA, Sanders G. Characteristics of individuals with Huntington disease in long-term care. Mov Disord. sept 1996;11(5):542-8.
218. Trejo A, Boll M-C, Alonso ME, Ochoa A, Velásquez L. Use of oral nutritional supplements in patients with Huntington's disease. Nutrition. sept 2005;21(9):889-94.
219. Popovic V, Svetel M, Djurovic M, Petrovic S, Doknic M, Pekic S, et al. Circulating and cerebrospinal fluid ghrelin and leptin: potential role in altered body weight in Huntington's disease. Eur J Endocrinol. oct 2004;151(4):451-5.
220. Cubo E, Rivadeneyra J, Armesto D, Mariscal N, Martinez A, Camara RJ, et al. Relationship between Nutritional Status and the Severity of Huntington's Disease. A Spanish Multicenter Dietary Intake Study. J Huntingtons Dis. 2015;4(1):78-85.
221. Rivadeneyra J, Cubo E, Polo CG, Mariscal N, Calvo S, Mateos A, et al. J14 Mediterranean Diet And Nutritional Composition Of Patients With Huntington's Disease. Spanish Multicenter Study Of The European Group For Huntington's Disease. J Neurol Neurosurg Psychiatry. 1 sept 2014;85(Suppl 1):A69-70.
222. Cubo E, Rivadeneyra J, Mariscal N, Armesto D, Mateos A, Camara R. The Relationship between Dietary Intake, Nutrition Status and Huntington's Disease Severity (P1.050). Neurology [Internet]. avril 2014;82(10 Supplement). Disponible sur: http://n.neurology.org/content/82/10_Supplement/P1.050.abstract
223. Reyes A, Cruickshank T, Nosaka K, Ziman M. Respiratory muscle training on pulmonary and swallowing function in patients with Huntington's disease: a pilot randomised controlled trial. Clin Rehabil. oct 2015;29(10):961-73.
